# African-specific polymorphisms in *Plasmodium falciparum* serine repeat antigen 5 in Uganda and Burkina Faso clinical samples do not interfere with antibody response to BK-SE36 vaccination

**DOI:** 10.3389/fcimb.2022.1058081

**Published:** 2022-12-16

**Authors:** Nobuko Arisue, Nirianne Marie Q. Palacpac, Edward H. Ntege, Adoke Yeka, Betty Balikagala, Bernard N. Kanoi, Edith Christiane Bougouma, Alfred B. Tiono, Issa Nebie, Amidou Diarra, Sophie Houard, Flavia D’Alessio, Odile Leroy, Sodiomon B. Sirima, Thomas G. Egwang, Toshihiro Horii

**Affiliations:** ^1^ Research Center for Infectious Disease Control, Research Institute for Microbial Diseases, Osaka University, Suita, Osaka, Japan; ^2^ Section of Global Health, Division of Public Health, Department of Hygiene and Public Health, Tokyo Women’s Medical University, Tokyo, Japan; ^3^ Department of Malaria Vaccine Development, Research Institute for Microbial Diseases, Osaka University, Suita, Osaka, Japan; ^4^ Department of Plastic and Reconstructive Surgery, University of the Ryukyus, Graduate School of Medicine and Hospital, Okinawa, Japan; ^5^ Makerere University School of Public Health, Kampala, Uganda; ^6^ Department of Tropical Medicine and Parasitology, School of Medicine, Juntendo University, Tokyo, Japan; ^7^ Centre for Malaria Elimination (CME) and Centre for Research in Infectious Diseases (CRID), Directorate of Research and Innovation, Mount Kenya University, Thika, Kenya; ^8^ Public Health Department, Institut National de Santé Publique/Centre National de Recherche et de Formation sur le Paludisme (INSP/CNRFP), Ouagadougou, Burkina Faso; ^9^ Groupe de Recherche Action en Santé (GRAS), Ouagadougou, Burkina Faso; ^10^ European Vaccine Initiative (EVI), Universitäts Klinikum Heidelberg, Heidelberg, Germany; ^11^ Sorekara-x consultant, Paris, France; ^12^ Med Biotech Laboratories, Kampala, Uganda

**Keywords:** BK-SE36 malaria vaccine, Plasmodium falciparum, serine repeat antigen 5, polymorphism, clinical trial

## Abstract

BK-SE36, based on *Plasmodium falciparum* serine repeat antigen 5 (SERA5), is a blood-stage malaria vaccine candidate currently being evaluated in clinical trials. Phase 1 trials in Uganda and Burkina Faso have demonstrated promising safety and immunogenicity profiles. However, the genetic diversity of *sera5* in Africa and the role of allele/variant-specific immunity remain a major concern. Here, sequence analyses were done on 226 strains collected from the two clinical trial/follow-up studies and 88 strains from two cross-sectional studies in Africa. Compared to other highly polymorphic vaccine candidate antigens, polymorphisms in *sera5* were largely confined to the repeat regions of the gene. Results also confirmed a SERA5 consensus sequence with African-specific polymorphisms. Mismatches with the vaccine-type SE36 (BK-SE36) in the octamer repeat, serine repeat, and flanking regions, and single-nucleotide polymorphisms in non-repeat regions could compromise vaccine response and efficacy. However, the haplotype diversity of SERA5 was similar between vaccinated and control participants. There was no marked bias or difference in the patterns of distribution of the SE36 haplotype and no statistically significant genetic differentiation among parasites infecting BK-SE36 vaccinees and controls. Results indicate that BK-SE36 does not elicit an allele-specific immune response.

## Introduction

Despite unprecedented gains in malaria control, progress has stalled in recent years. Children under 5 years old in sub-Saharan Africa continue to shoulder a disproportionate share of the malaria burden (Weiss et al, 2019; WHO, 2021). In a historic move, the World Health Organization (WHO) endorsed the first-ever malaria vaccine, RTS,S (Maxmen, 2021). RTS,S, based on the circumsporozoite protein (CSP), has a modest efficacy of 36% against malaria over 4 years of follow-up (Laurens, 2020). After pilot implementation studies that confirmed safety as well as its feasible deployment, modeling studies show that 4 doses will avert 116,480 cases of clinical malaria and 484 deaths per 100,000 vaccinated children (Laurens, 2020). The contribution of a more effective second-generation malaria vaccine cannot be overemphasized.

One major impediment to the elusive goal of a highly efficacious vaccine is the polymorphic nature of antigens that alter epitope antibody responses, leading to low or limited vaccine efficacy (Palacpac and Horii, 2020). In phase 3 trials, RTS,S had a better overall efficacy at protecting against malaria caused by vaccine-strain than non-vaccine strain CSP in 5–17-month-old children (Neafsey et al., 2015). Efficacy was approximately 10–15% lower against non-vaccine (mismatched) type parasite infections. Other candidate vaccines based on polymorphic merozoite surface protein 1 (MSP: FMP1/AS02) (Ogutu et al., 2009) and apical membrane antigen 1 (AMA1: FMP2.1/AS02_A_, AMA1-C1, AMA1-FVO) (Takala et al., 2009; Thera et al., 2011) did not show significant overall protection in proof-of-concept trials but had allele-specific efficacy against clinical malaria. Sequence analyses of *ama1* genes obtained in a phase 2 trial revealed that the AMA1 vaccine reduced the risk of clinical malaria only when the infecting parasite had identical amino acid residues to the vaccine type at a key position in the AMA1 cluster 1 loop (Ouattara et al., 2013). These studies highlight the need for molecular epidemiological studies to identify and determine the roles of polymorphisms in natural populations and during vaccine trials, particularly in cases of genetic and antigenic diversity and vaccine failure.

**Figure 1:**
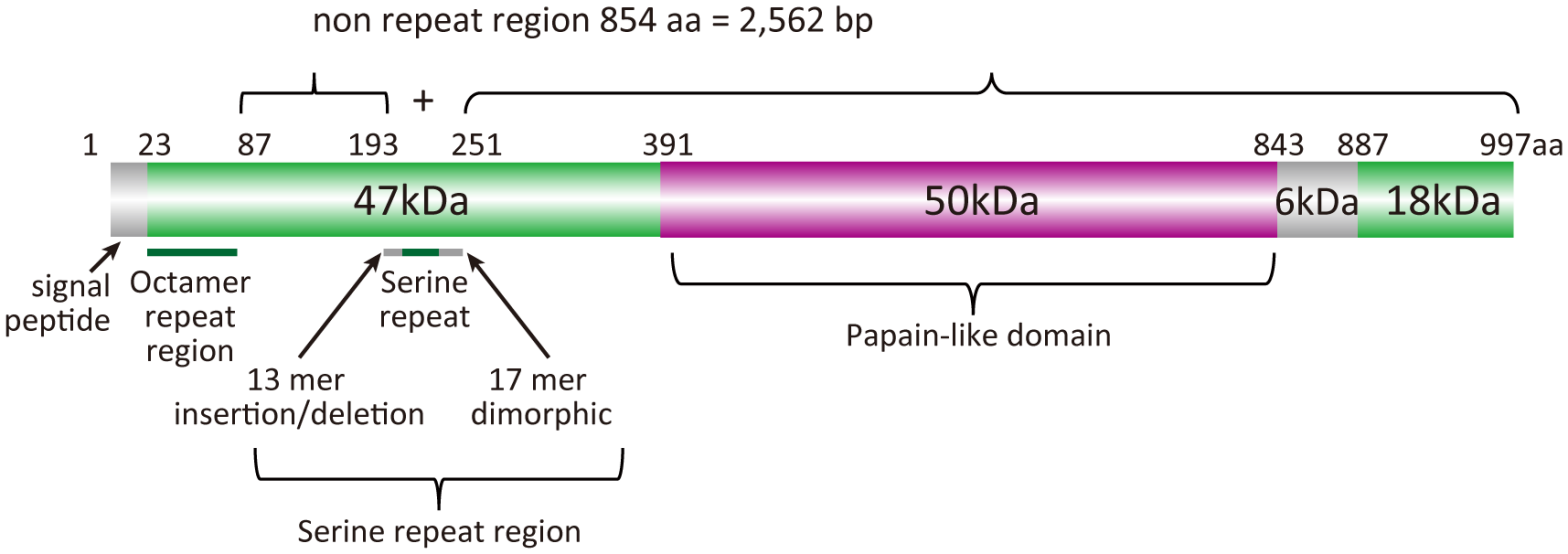
Schematic representation of the SERA5 structure. *Plasmodium falciparum* 3D7 strain was used as a reference for the illustration.

BK-SE36 is a formulation based on the serine repeat antigen 5 (SERA5) of *Plasmodium falciparum*. SERA5 is a blood-stage antigen (Arisue et al., 2020) expressed during the late trophozoite and schizont stages as a 120-kDa precursor and secreted into the parasitophorous vacuole after removal of the signal peptide (Debrabant et al., 1992). The protein is cleaved by subtilisin-like serine protease 1 into 47-, 56-, and 18-kDa fragments (Yeoh et al., 2007) (**Figure 1**). The 47-kDa fragment is linked to the 18-kDa fragment *via* a disulfide bond and localizes to the merozoite surface (Li et al., 2002). The 56-kDa fragment containing the papain-like catalytic domain is further cleaved by an unknown protease to 50- and 6-kDa fragments just before parasite egress (Li et al., 2002; Yeoh et al., 2007; Stallmach et al., 2015). SE36 is identical to the 47-kDa fragment (P47) except for the serine repeats which were removed to improve the hydrophilicity of the protein.

The N-terminal fragment, SE36, was selected for clinical development based on the findings that (i) antibodies against the P47 domain (SE36 with the serine repeat region) inhibited parasite growth *in vitro* (Fox et al., 1997; Pang and Horii, 1998; Pang et al., 1999; Aoki et al., 2002; Yagi et al., 2014); (ii) seroepidemiological studies in malaria-endemic areas showed a negative correlation between parasitemia and anti-P47 antibody titers (Okech et al., 2001; Horii et al., 2010); and (iii) high anti-SE36 antibody titers were associated with protection from severe malaria outcomes (Okech et al., 2006; Owalla et al., 2013). BK-SE36, which is SE36 adsorbed to aluminum hydroxide gel, is a malaria vaccine candidate currently in clinical trials. The phase 1b randomized trial and follow-up study in Uganda, conducted in 2010–2012, showed that the vaccine was safe and immunogenic; and the 6–20-year-olds that received the vaccine had a reduced risk of time-to-first episodes and all episodes of high parasitemia (>5000 parasites/µL) and fever at 130–365 days post-second vaccination (Palacpac et al., 2013). A clinical trial in Burkina Faso with 12–60-month-old children was also completed (Bougouma et al., 2022).

Epitope mapping of the SE36 region in SERA5 using serum samples from a malaria endemic area indicated that the intrinsically unstructured N-terminal octamer repeat region possessed inhibitory epitopes (Yagi et al., 2014). Sequence analyses of SERA5 genes using 445 P*. falciparum* isolates collected from nine endemic countries in Africa, Southeast Asia, Oceania, and South America revealed variations in the (i) number and motifs of octamer amino acid units in the SERA5 N-terminal domain, (ii) number of serine repeats and polymorphisms in the 13-mer insertion/deletion region and 17-mer dimorphic sequence flanking these repeats, and (iii) area-specific single-nucleotide polymorphisms (SNPs) in non-repeat regions of the gene (Tanabe et al., 2012). Thus, although no strong signature for positive selection was detected in the non-repeat sequence regions (2,562 bp), protective epitopes were located in predicted disordered regions of the protein. Whether these polymorphisms are involved in allele-specific immunity remains unclear. Furthermore, information on SERA5 polymorphisms in *P. falciparum* isolates circulating in Africa is limited. Here, the different features of the polymorphism in *sera5* and other antigen and housekeeping genes were examined. Patterns of genetic variation were compared for parasites isolated within Africa and for those vaccinated with BK-SE36 and saline. Variations in *sera5* did not appear to influence the effectiveness of BK-SE36 to elicit an immune response.

## Materials and methods

### Ethics approval

Ethical approval for the Ugandan trial and follow-up study were obtained from the ethical institutional review committees of Med Biotech Laboratories (Ref# IRB-00003990-MBL-BIOMEDICAL, IRB-00003995-MBL-BIOMEDICAL), Uganda National Council for Science and Technology (Ref# HS635, HS866), Osaka University (Ref# 20-3, 287), and Research Foundation for Microbial Diseases of Osaka University.

Approval for the Burkina Faso trial was obtained from Comité d’Éthique pour la Recherche en Santé du Burkina Faso (Ref# 2014-12-144), Comité Institutionnel de Bioéthique du INSP/CNRFP (previous name: CNRFP) (Ref# N°2014/071/MS/SG/CNRFP/CIB, N°2016/000008/MS/SG/CNRFP/CIB), Agence Nationale de Régulation Pharmaceutique (ANRP, previous name: Direction Générale de la Pharmacie, du Médicament et des Laboratoires [DGPML], Ref# N°2015_658/MS/CAB), Scientific Committee/Institutional Review Committee of the Research Institute for Microbial Diseases (Ref# 26), Osaka University (Ref# 574); and London School of Hygiene and Tropical Medicine Research Ethics Committee (Ref# 9175).

Consent, either by signature or thumbprint, was obtained from the children and/or children’s parents or guardians prior to sampling and other trial-related procedures. In Uganda, for subjects 8–17 years old, assent by the child took precedence over consent from the parents or guardians.

All studies were conducted in compliance with the protocol, International Council for Harmonisation of Technical Requirements for Pharmaceuticals for Human Use (ICH), Good Clinical Practices, the Declaration of Helsinki 2013, and country-specific laws and regulations.

### Clinical trial in Uganda

The details of the randomized, single-blind, safety and immunogenicity phase 1b trial (Current Controlled trials ISRCTN71619711 https://doi.org/10.1186/ISRCTN71619711) and follow-up study in Lira, northern Uganda have been previously described (Palacpac et al., 2013; Yagi et al., 2016). The clinical trial site, Lira Medical Centre, is in a region with intense transmission and bimodal rainfall pattern from April to May and September to October (Proietti et al., 2011). Blood samples and data on malaria incidence were obtained from healthy children and young adults aged 6–20 years old (n = 84). Participants in 3 age cohorts (6–10, 11–15, and 16–20 years old) were randomized to be administered twice with either half- (0.5 mL, n=11) or full- (1.0 mL, n=6) dose BK-SE36 or saline (n=6). Subcutaneous vaccination was performed 21-days apart. During the follow-up study (130–440 days post-second vaccination), 50 additional children (as unvaccinated controls) were recruited. Recruitment of the additional control group used the same inclusion and exclusion criteria as in the clinical trial and was age-, gender-, and locality-matched as much as possible. The paired control participants visited the trial center on the same scheduled day as trial participants to undergo assessments for vital signs, physical examination, monthly questionnaire, and blood smear. Blood samples for filter blots, thick and thin blood smears, were obtained at 28-day intervals (active surveillance) or whenever the child was sick (passive surveillance). In this study, any blood smear-positive samples (any parasitemia) were used for analyses.

### Clinical trial in Burkina Faso

The Burkina Faso trial was a double-blind, randomized, controlled, age de-escalation safety and immunogenicity trial and follow-up study conducted in Banfora, south western Burkina Faso (Pan African Clinical Trials Registry: PACTR201411000934120; https://pactr.samrc.ac.za/TrialDisplay.aspx?TrialID=934 ) (Bougouma et al., 2022). The trial site, Unité de Recherche Clinique de Banfora, is in a region where transmission occurs throughout the year with a peak for clinical malaria occurring within the 4 months (June–September) of the rainy season of May–November (Tiono et al., 2014). Samples and data of clinical malaria episodes were obtained from healthy children aged 12–60 months (n = 108). Participants in two age cohorts (12–24 and 25–60-months-old) were randomized for administration of Synflorix^®^ (0.5 mL, control arm, n = 18); or BK-SE36 (1.0 mL) *via* either the subcutaneous route (n = 18), or the intramuscular route (n = 18). Two administrations were performed 28 days apart, and a third dose was administered at week 26 (Day 182). The control group was vaccinated with Synflorix^®^ at Days 0 and 182, and with saline at Day 28 to comply with the manufacturer’s recommendation of at least a 2-month interval between the 2 primary Synflorix^®^ doses while ensuring that the trial was performed in a double-blinded manner. Clinical malaria episodes, defined as ≥5000 parasites/µL + tympanic temperature ≥38°C, from Day 56 (4 weeks after dose 2) until the final visit (Day 477, 42 weeks after dose 3) were assessed. Two thick and thin blood smears were prepared for malaria diagnosis and blood samples spotted onto Whatman™ 903 Protein Saver Card (GE Healthcare Life Sciences, MA, USA) were dried and stored until use.

### DNA preparation, SERA5 gene amplification, and nucleotide sequencing

The BioRobot EZ1 DNA investigator kit (QIAGEN, Hilden, Germany) was used to extract parasite DNA from the filter paper blood spots. Four pieces of a 3-mm-diameter circle, corresponding to a 20-μL volume of blood, were punched out from 1-2 blood spots for downstream reactions. The extracted DNA was resuspended in 50 μL TE solution, and stored at -20°C until use.

Exon regions II-IV of the SERA5 gene, covering approximately 3.3 kb, were amplified using specific primers sera5-F1 and sera5-R1 (**Supplementary Table S1**). Exon I encoding the signal peptide was not analyzed because of sequencing difficulties. Amplification was carried out in a 25-μL reaction mixture containing 0.4 μM each of forward and reverse primers, 0.4 mM each of dNTP, 0.5 units of KOD FX Neo polymerase (TOYOBO, Osaka, Japan), 12.5 μL of 2x PCR buffer, and 1 μL of genomic DNA solution. PCR conditions were as follows: 95°C for 2 min, 35 cycles of 95°C for 15 sec, 59°C for 30 sec, and 68°C for 2 min. A 2-μL aliquot of the PCR product was used as template for a second PCR amplification in a 25-μL reaction mixture using the primers sera5-F2 and sera5-R2 (**Supplementary Table S1**) under the same thermocycler conditions. In case of failure with the first set of primers, other primer pairs were also tested: sera5-F1 and sera5-R1 for the 1st PCR and sera5-F3 and sera5-R2 for the 2nd PCR or sera5-F2 and sera5-R1 for the 1st PCR and sera5-F3 and sera5-R2 for the 2nd PCR. PCR products were purified using the QIAquick PCR Purification kit (QIAGEN) according to the manufacturer’s instructions. Purified DNA fragments were eluted in 30 μL TE. Optical density was measured with a NanoDrop (Thermo Fisher Scientific, Waltham, MA, USA) and the DNA concentration was adjusted to 0.026 μg/μL using TE. At this concentration, 1 μL was suitable for performing one sequencing reaction.

DNA sequencing was performed directly using the BigDye^®^ Terminator v3.1 Cycle Sequencing Kit and 3130xI Genetic Analyzer (Applied Biosystems, Foster City, CA, USA). Sequencing primers were designed to cover target regions in both directions (**Supplementary Table S1**). If inconsistencies were obtained after two independent amplifications, a third round of PCR/sequencing reaction was performed. Only isolates showing a single genotype infection, without overlapping peaks on the electropherograms, were used for further analysis. To compare the nucleotide diversity of SERA5 with other antigens and housekeeping genes, the sequences of apical membrane antigen 1 (*ama1*), circumsporozoite protein (*csp*), Ca2+-transporting ATPase (*serca*), and adenylosuccinate lyase (*adsl*) genes were determined using the Ugandan isolates. The PCR and sequencing strategies were the same as those used for *sera5* except that the PCR conditions were adjusted to be suitable for each gene. The PCR sequencing primers are listed in (**Supplementary Table S1**).

### Accession numbers

The newly determined nucleotide sequences in this study have been deposited in the DNA Data Bank of Japan (accession nos. LC580441– LC581218). Samples from a previous study were used to compare polymorphisms found in Africa. The Tanzania and Ghana isolates were described previously (Tanabe et al., 2010). Briefly, Tanzania blood samples (n = 55) were collected from residents of Nyamisati village in the Rufiji River Delta, eastern coastal Tanzania in February and March 1993, 1998, and January 2003. Asymptomatic donors had a mean age of 14.2 years (range, 1–78), 16.8 years (range, 1–63), and 13.8 years (range, 10–19) in 1993, 1998, and 2003, respectively. Ghana isolates (n = 33) were collected during malaria surveys in 0–15-year-old children from three villages near Winneba (Okyereko, Mpota, and Apam), a western coastal region, in November 2004. The accession numbers of *sera5*, *ama1*, *csp*, *serca*, and *adsl* are summarized in **Supplementary Table S2**.

### Sequence alignment and genetic analyses

The nucleotide sequences of *sera5* were aligned using CLUSTALW implemented in GENETYX^®^ ver. 15 (GENETYX Corporation, Tokyo, Japan) with manual corrections. According to our previous analyses (Tanabe et al., 2012), the SERA5 sequence was categorically divided into an octamer repeat (OR) region, serine repeat (SR) region, and non-repeat regions (2,562 bp) (**Figure 1**). The number of haplotypes, haplotype diversity (Hd), and nucleotide diversity (*θ*_π_) were calculated using DnaSP v5.10.01 (Librado and Rozas, 2009). The difference between the numbers of synonymous substitutions per synonymous site (dS) and nonsynonymous substitutions per nonsynonymous site (dN) was calculated by the Nei and Gojobori method (Nei and Gojobori, 1986) with Jukes and Cantor correction as implemented in MEGA X (Kumar et al., 2018). The statistical significance of the difference between dN and dS was estimated with MEGA Z-test. If dN was greater than dS, positive selection was predicted. Genetic differentiation of SERA5 among the parasite population was examined using *Fst*, the Wright’s fixation index (Wright, 1965) of inter-population variance in allele frequencies. Pairwise *Fst* between parasite populations was calculated using Arlequin v3.5 (Excoffier and Lischer, 2010). These programs had built-in statistical analyses which were performed automatically, however, when statistical analyses were not supported, Mann-Whitney test for differences between two independent groups, the Kruskal-Wallis test for differences between multiple groups, and the chi-square (χ2) test for proportions between group were used.

## Results

### Sample description

In the trial conducted in Uganda, of the 247 infection events recorded, sequence information from 172 infections was obtained (75 were mixed infections and excluded from further analyses). Parasitemia ranged from 16 to 151,680 parasites/µL and 4 samples had *P. falciparum* gametocyte. Of the 172 available sequences, 33 were from subjects who had only one infection and 66 were from participants who had at least two infections during the follow-up period. In summary, 77 isolates were from the BK-SE36 vaccine arm and 95 from the control group. The samples were collected from March 2011 to February 2012, covering 2 malaria transmission seasons.

For the Burkina Faso trial, of 78 clinical malaria events, sequence information was obtained from 54 cases (24 were mixed infections and excluded from further analyses). Parasitemia ranged from 6,527 to 253,900 parasites/µL. Of the 54 available sequences, 29 sequences were from subjects who had only one infection and 10 were from subjects who had at least two infections during the follow-up period. Twenty-nine sequences were from the BK-SE36 vaccine arm and 25 from the control group. Samples were obtained from July 2015 to January 2017, covering the rainy season of May–November. Each subject had a total follow-up period of 16 months.

### Comparison of SERA5 polymorphism with major antigen genes and housekeeping genes

**Figure 2:**
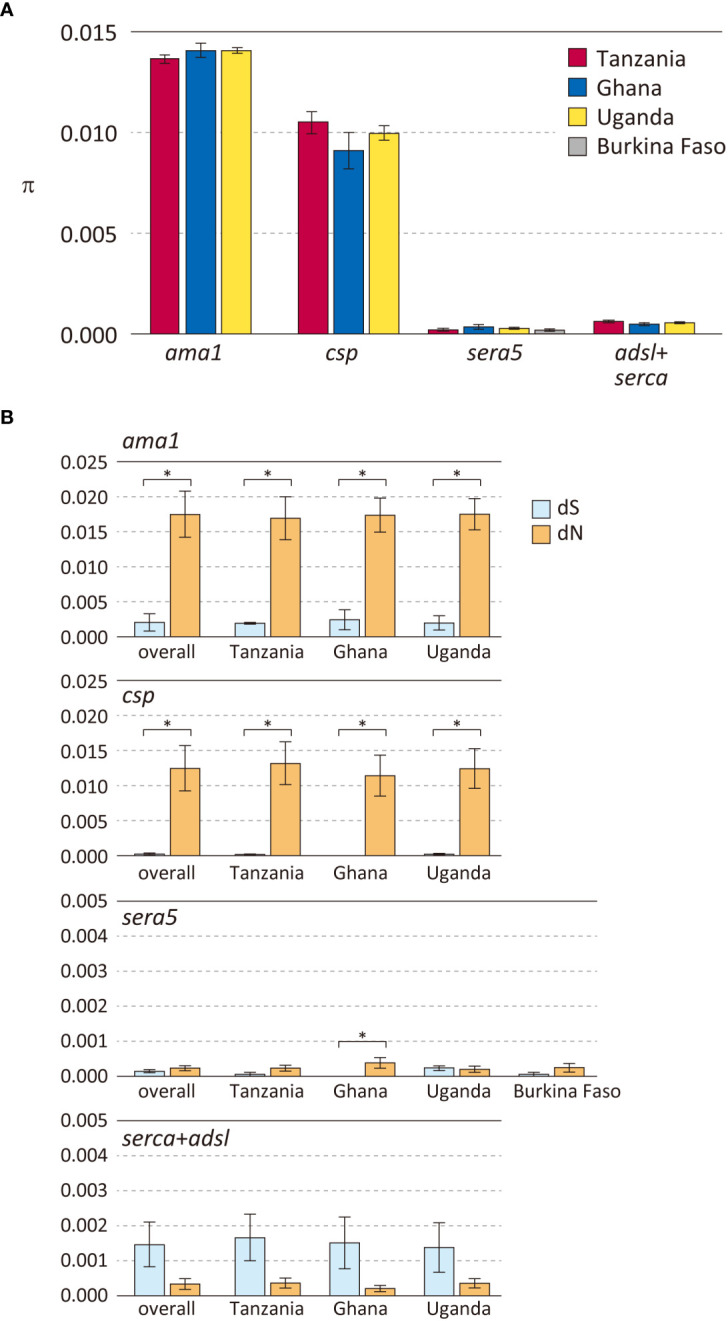
Polymorphisms in three *P. falciparum* vaccine candidate genes and housekeeping genes in parasite populations from Africa. **(A)** Nucleotide diversities (*θ*_π_) are shown. Error bars reflect standard deviation. **(B)** Number of synonymous substitutions per synonymous site (dS) and number of non-synonymous substitutions per non-synonymous site (dN) are shown. Error bars reflect the standard error. For details, see **Supplementary Table S3**. Differences between dS and dN that were significant (dN>dS, *p* < 0.01), denoting positive selection, are indicated in asterisk.

*P. falciparum* isolates from the four African countries were used to compare polymorphisms in 3 antigen genes (*sera5*, *ama1* and *csp*) and 2 housekeeping genes (*serca* and *adsl*) (**Figure 2**, **Supplementary Table S3**). As expected, the degree of polymorphism varied between antigen-coding genes and housekeeping genes. Notably, the level of polymorphism in all African isolates within each gene was similar. The Hd of the three antigen genes *ama1*, *csp*, and *sera5* (**Supplementary Table S4A**) were extremely high (close to 1.0), demonstrating that most sequences were distinct from each other. The Hd of the concatenated housekeeping genes of *adsl* and *serca* (*adsl*+*serca*) was slightly lower than that of the antigen genes. In the antigen genes, the number of sequence variations was almost the same in both the nucleotide and amino acid sequences; however, in the housekeeping genes, the amino acid sequence showed far fewer variations compared to the nucleotide sequences. Nucleotide substitutions in the antigen genes mainly occurred at non-synonymous sites which resulted in amino acid changes, whereas in housekeeping genes, nucleotide substitutions tend to occur at synonymous sites without amino acid changes.

Nucleotide diversity was estimated as *θ_π_*, the average pairwise nucleotide difference (**Figure 2A**). The *θ_π_* of the antigen genes *ama1* and *csp* were very high, whereas that of the antigen gene *sera5* was much lower and comparable to the concatenated gene sequences of *adsl+serca*. Among the three antigen genes, *ama1* and *csp* showed a signature of diversifying selection with a significant excess of dN over dS (**Figure 2B**). The high values of *θ_π_* and dN in *ama1* and *csp* indicate large differences between the sequences, and dN>dS is likely due to host immune evasion. In contrast, the values of *θ_π_*, dN, and dS of *sera5* were much lower than those of *ama1* and *csp*. Although dN was significantly larger than dS in *sera5* from the Ghana isolates, the differences between the amino acid sequences were small compared to either *ama1* or *csp* (**Figure 2B**). For each of these genes, analyses for *θ_π_*, dS, and dN were performed to compare Hd with (**Supplementary Table S4A**) or without regions containing insertions and deletions (**Supplementary Table S4B**). The comparison provides insight into the various processes involved in generating alleles in antigen genes. The excluded regions would refer to the ‘NANP’ repeat region in *ama1*; the eight-mer amino acid repeat units in *csp*; the serine repeat region, the 13-mer insertion/deletion region, and the 17-mer dimorphic region in *sera5*; and the asparagine repeat region in *adsl*+*serca.* When Hd of *sera5* was analyzed using the same dataset which excluded insertions and deletions, the value of Hd was much smaller than that obtained using full-length *sera5* (**Supplementary Table S4**). The Hd of *ama1* was similar, as there were no insertions or deletions in the gene. The Hd of *csp* was also similar despite the exclusion of NANP repeat regions. The lower Hd of *sera* when regions of insertions and deletions are removed suggests that sequence variations in *sera5* are mainly introduced by recombination rather than by point mutation. In *ama1*, all polymorphisms are produced by point mutation, whereas in *csp*, both point mutation and recombination resulting in different numbers of NANP units act to generate sequence variations.

### Sequence comparison of SE36 region in SERA5

**Figure 3:**
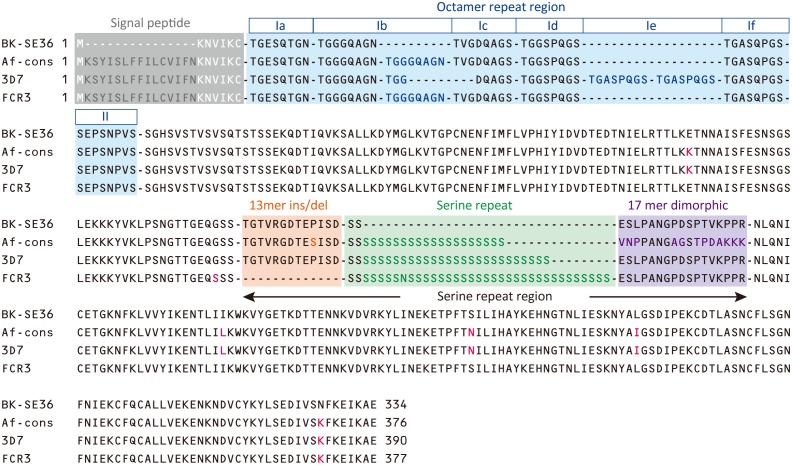
Sequence comparison of SE36 region in SERA5. The African consensus sequence (Af-cons) of the SE36 region was derived from sequence comparison of 314 SERA5 sequences from four African countries. Amino acid sequences of the corresponding SE36 region were compared among vaccine-type variants (the vaccine candidate based on *P. falciparum* Honduras 1), 3D7 and FCR3 strains, and Af-cons. Characteristic regions are shaded in color: signal peptide, octamer repeat region, serine repeat region. Amino acids that differ from vaccine-type SE36 are shown in red font. Octamer repeat sequence was classified into Ia to If and II according to a previous study (Tanabe et al., 2012).

The consensus sequence of SE36 in SERA5 was inferred from 314 sequences obtained from four African countries: Uganda (n = 172), Burkina Faso (n = 54), Tanzania (n = 55), and Ghana (n = 33). The consensus sequence in African isolates (Af-cons) was aligned with the SE36 sequence based on Honduras-1 and two representative laboratory strains, 3D7 and FCR3. The BK-SE36 candidate vaccine was designed with reference to the SERA5 sequence of the Honduras-1 strain (Sugiyama et al., 1996). As shown in **Figure 3**, there were several sequence mismatch regions, particularly between the vaccine-type SE36 variant and Af-cons.

At the N-terminal, of the 314 SERA5 sequences, there were 76 variations in the OR region (**Supplementary Figure S1**). The number of octamer units varied widely: seven octamer units (23 OR variations) were detected in 196 SERA5 sequences across Africa (62.4%), eight octamer units (16 OR variations) were found in 47 sequences (15.0%), and six octamer units (8 OR variations) were found in 14 sequences (4.5%). In all four African countries, seven octamer units were the most frequently detected. The number of OR units detected from SERA5 sequences was not significantly different among the four countries (*p* > 0.05, Kruskal-Wallis test). Vaccine-type SE36 has six octamer units, made up of one Ib subunit; and an identical OR sequence was found only in 5 *P. falciparum* isolates (1.6%): 4 from Uganda and 1 isolate from Ghana (**Table 1**; **Supplementary Figure S1**). The seven octamer units found predominantly in Af-cons contain two Ib subunits (**Figure 3**).

**Table 1:** Number and frequency of octamer units found in SERA5 sequences from 314 African isolates.

No of octamer unit	total n = 314	Uganda n = 172	Burkina Faso n = 54	Tanzania n = 55	Ghana n = 33
2	2 (0.6)	N.D.	N.D.	1 (1.8)	1 (3.0)
5	1 (0.3)	1 (0.6)	N.D.	N.D.	N.D.
6	14 (4.5)	7 (4.1)	5 (9.3)	1 (1.8)	1 (3.0)
7	196 (62.4)	119 (69.2)	23 (42.6)	38 (69.1)	16 (48.5)
8	47 (15.0)	17 (9.9)	15 (27.8)	5 (9.1)	10 (30.3)
9	17 (5.4)	9 (5.2)	4 (7.4)	4 (7.3)	N.D.
10	6 (1.9)	2 (1.2)	2 (3.7)	N.D.	2 (6.1)
11	10 (3.2)	4 (2.3)	4 (7.4)	2 (3.6)	N.D.
12	10 (3.2)	7 (4.1)	1 (1.9)	N.D.	2 (6.1)
13	4 (1.3)	1 (0.6)	N.D.	3 (5.5)	N.D.
14	4 (1.3)	3 (1.7)	N.D.	1(1.8)	N.D.
15	2 (0.6)	1 (0.6)	N.D.	N.D.	1 (3.0)
35	1 (0.3)	1 (0.6)	N.D.	N.D.	N.D.

The frequency is shown as a percentage in parentheses.

For details, see **Supplementary Figure S1**.

N.D. not detected.

The SR region has three distinct sequence components: a 13-mer insertion/deletion, stretch of serine repeats, and a 17-mer dimorphic region (Morimatsu et al., 1997; Tanabe et al., 2012). A total of 69 amino acid sequence variations was identified in the SR region (**Supplementary Figure S2**). The 13-mer deletion in the SR region of the FCR3 strain (**Figure 3**) was observed in Oceania and South America, but not in Africa (Tanabe et al., 2012). In vaccine-type SE36 and Af-cons, the 13-mer sequence contained a serine/proline variation at position 203: a proline residue was found in the vaccine-type and 3D7 strain but a serine residue was mostly found in all isolates from the four African countries. SERA sequences did not differ significantly among the four African countries in terms of serine/proline ratios at position 203 (*p* > 0.05, χ^2^-test) (**Table 2**).

**Table 2:** Frequency of serine/proline residues at position 203 in the 13-mer insertion/deletion region of SERA5.

		position 203	
	n	Serine	Proline
Uganda	172	125 (72.7)	47 (27.3)
Burkina Faso	54	41 (75.9)	13 (24.1)
Tanzania	55	35 (63.6)	20 (36.4)
Ghana	33	18 (54.5)	15 (45.5)

The amino acid position is numbered after the 3D7 sequence.

The frequency is shown as a percentage in parentheses.

For the stretch of serine tandem repeats from which SERA5 was named, vaccine-type SE36 contains only two serine residues (**Figure 3**). This stretch of serine tandem repeats was mostly deleted from the vaccine construct to improve the hydrophilicity of the protein and make it amenable for large-scale Good Manufacturing Practices (GMP) production (Palacpac et al., 2011). Among the 314 African isolates, the number of poly-serine residues varied from 5 to 43, with 21 repeats as the most common in all 4 African countries (**Table 3**). The difference in the number of serine residues among the four countries was statistically significant (*p* < 0.05, Kruskal-Wallis test). Notably, the amino acids isoleucine, asparagine, glycine, and arginine were also found in the serine stretch in eight of the 314 African isolates (**Supplementary Figure S2**).

**Table 3:** Number of serine repeats/amino acid residues in the stretch of serine tandem repeats of the SERA5 SR region.

No. of amino acid	Uganda	Burkina Faso	Tanzania	Ghana
	n = 172	n = 54	n = 55	n = 33
5	1 (0.6)	N.D.	1 (1.8)	1 (3.0)
11	1 (0.6)	N.D.	1 (1.8)	N.D.
15	7 (4.1)	3(5.6)	6 (10.9)	2 (6.1)
17	3 (1.7)	N.D.	N.D.	N.D.
19	1 (0.6)	1 (1.9)	1 (1.8)	N.D.
21	44 (25.6)	21 (38.9)	16 (29.1)	8 (24.2)
23	21 (12.2)	10 (18.5)	6 (10.9)	3 (9.1)
25	16 (9.3)	7 (13.0)	4 (7.3)	2 (6.1)
27	12 (7.0)	3 (5.6)	4 (7.3)	1 (3.0)
29	7 (4.1)	1 (1.9)	3 (5.5)	4 (12.1)
31	43 (25.0)	5 (9.3)	11 (20.0)	5 (15.2)
33	8 (4.7)	3 (5.6)	1 (1.8)	3 (9.1)
35	3 (1.7)	N.D.	N.D.	2 (6.1)
37	1 (0.6)	N.D.	N.D.	N.D.
39	2 (1.2)	N.D.	N.D.	N.D.
41	2 (1.2)	N.D.	N.D.	1 (3.0)
43	N.D.	N.D.	1 (1.8)	1 (3.0)

The frequency is shown as a percentage in parentheses.

N.D. not detected.

Just after the poly-serine repeat is a 17-mer dimorphic region (**Figure 3**) containing four major sequence variations (Tanabe et al., 2012): ‘VNPPANGAGSTPDAKKK’ (Type I), ‘ESLPANGPDSPTVKPPR’ (Type IV), and recombination forms ‘VNPPANGAGSTPDAKKR` (Type II) and ‘VNPPANGPDSPTVKPPR’ (Type III). Vaccine-type SE36 has the Type IV sequence, whereas all African isolates had Type I as dominant sequence (**Table 4**). For the four African countries, the ratio of Type I to Type IV sequence was significantly different (*p* < 0.01, χ2-test); and notably, no Type II and Type III sequences were found in Burkina Faso isolates. There were also sequence variations in Type IV. Substitutions at position 241 of proline to leucine and position 244 of proline to leucine were found in one Ugandan and Tanzanian isolate, respectively (**Supplementary Table S2**, **Supplementary Figure S2**).

**Table 4:** Frequency of Type I to Type IV sequence types in the 17-mer dimorphic sequence of SERA5 SR region.

		17 mer dimorphic sequence			
	n	Type I	Type II	Type III	Type IV
Uganda	172	90 (52.3)	10 (5.8)	37 (21.5)	35 (20.3)
Burkina Faso	54	46 (85.2)	N.D.	N.D.	8 (14.8)
Tanzania	55	21 (38.2)	9 (16.4)	11 (20.0)	14 (25.5)
Ghana	33	21 (63.6)	1 (3.0)	1 (3.0)	10 (30.3)

The frequency is shown as a percentage in parentheses.

N.D. not detected.

**Figure 4:**
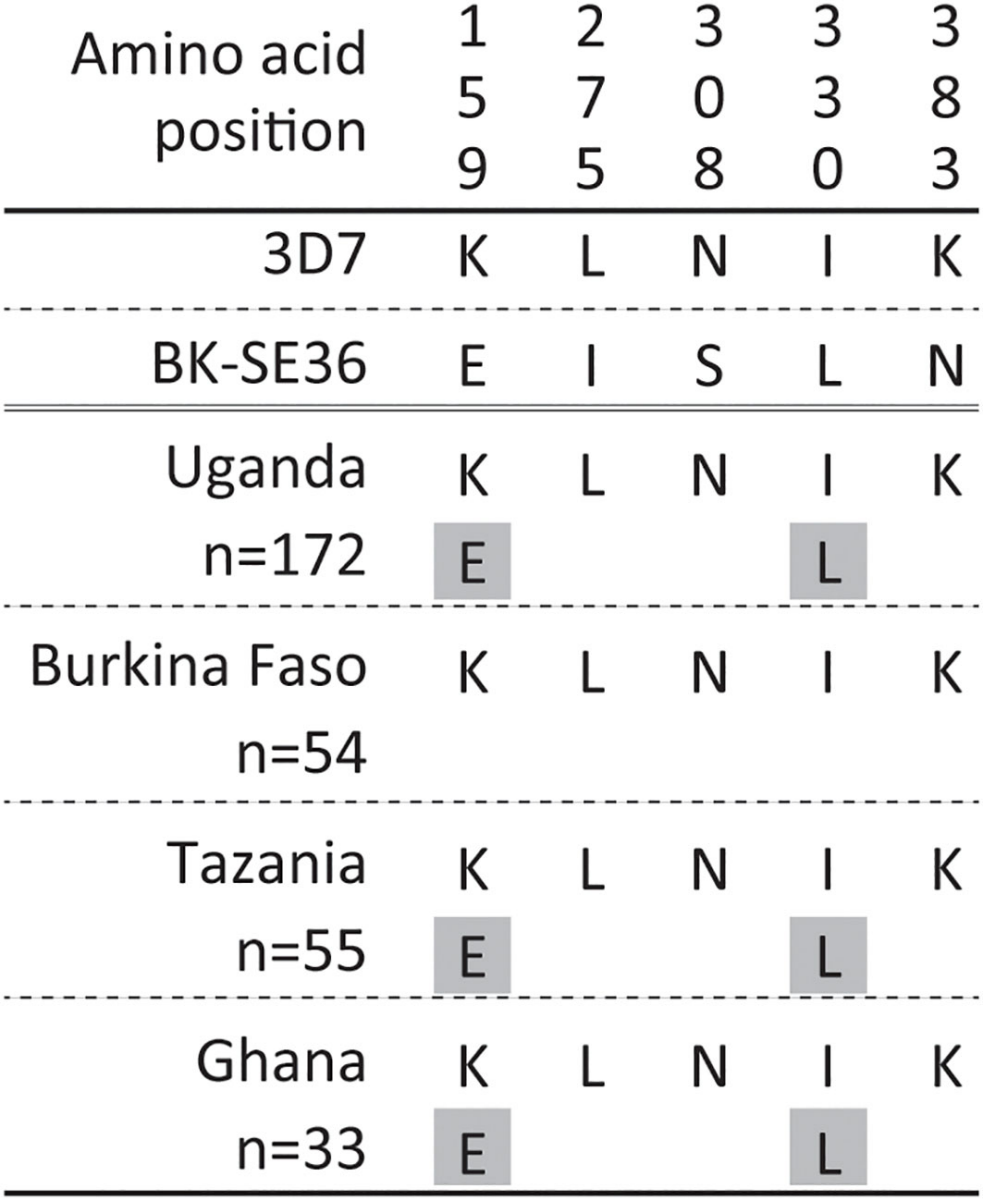
Amino acid variation in vaccine-type SE36 with the octamer and serine repeat regions removed from the analyses. Amino acid positions are numbered after the *P. falciparum* 3D7 sequence. The gray background shows a minor allele with only one allele found in each population.

In non-repeat regions, amino acid mismatches were observed in Af-cons at five positions (**Figure 3**). Those were at positions 159, 275, 308, 330, and 383 of the SERA5 amino acid sequence of *P. falciparum* strain 3D7. Among them, glutamic acid at position 159 and leucine at position 330 were also found in Uganda (n=1), Tanzania (n=1), and Ghana (n=1) isolates, although their frequencies were very low (**Figure 4**). In the vaccine-type SE36, at positions 275, 308, and 383, isoleucine, serine, and lysine were found, whereas African isolates exclusively showed leucine, asparagine, and lysine, respectively.

According to the above results, there are multiple differences between the vaccine-type SE36 and the corresponding SERA5 sequence from African isolates. The occurrence of identical sequences to the vaccine-type variant was rare.

### Sequence variation in SE36 of *P. falciparum* clinical isolates obtained from vaccinated and control groups

If BK-SE36 confers allele-specific immune response or shows efficacy targeting only vaccine-type parasites or closely related variants, then vaccinated individuals may be selectively infected with *P. falciparum* strains whose SERA5 sequences are less identical/heterologous to vaccine-type variant. Additionally, if sequence-dependent selection occurs, the haplotype diversity of SERA5 in the vaccinated group may be lower than that of SERA5 in the control group. To explore these possibilities, the haplotype diversity of SE36; OR and SR regions of SERA5 were compared between isolates from the BK-SE36 arm and isolates from the control arm in Uganda and Burkina Faso trials (**Supplementary Table S5**). The haplotype diversity of SERA5 was similar between the vaccinated and control groups at the two trial sites. In the OR and SR regions, haplotype diversity was slightly higher in the vaccinated group than in the control group.

Four Ugandan isolates showed the same amino acid sequence as the OR region of vaccine-type SE36; three were from the BK-SE36 vaccine arm and one from the control arm. No isolate from Burkina Faso showed an identical OR sequence (**Supplementary Figure S1**). In terms of the number of OR repeat units, seven Ugandan isolates and five Burkina Faso isolates had six OR units, similar to the vaccine-type variant (**Table 5**). There was no significant difference in the frequency of the six OR units between the vaccinated and control groups, both in Uganda and Burkina Faso isolates (*p* > 0.05, χ2-test). As inferred above, from the African samples, seven OR units were the most common in both Uganda and Burkina Faso isolates, although overall no marked differences in OR frequencies can be seen between the vaccinated and control groups.

**Table 5:** Number and frequency of octamer units found in the SERA5 sequence from clinical trial sites.

No. of octamer unit	Uganda			Burkina Faso		
	total	vaccinated	controls	total	vaccinated	controls
	n = 172	n = 77	n = 95	n = 54	n = 29	n = 25
5	1 (0.6)	1 (1.3)	N.D.	N.D.	N.D.	N.D.
6	**7 (4.1)**	**3 (3.9)**	**4 (4.2)**	**5 (9.3)**	**3 (10.3)**	**2 (8.0)**
7	119 (69.2)	55 (71.4)	64 (67.4)	23 (42.6)	14 (48.3)	9 (36.0)
8	17 (9.9)	8 (10.4)	9 (9.5)	14 (25.9)	8 (27.6)	6 (24.0)
9	9 (5.2)	5 (6.5)	4 (4.2)	5 (9.3)	1 (3.4)	4 (16.0)
10	2 (1.2)	1 (1.3)	1 (1.1)	2 (3.7)	1 (3.4)	1 (4.0)
11	4 (2.3)	1 (1.3)	3 (3.2)	4 (7.4)	1 (3.4)	3 (12.0)
12	7 (4.1)	2 (2.6)	5 (5.3)	1 (1.9)	1 (3.4)	N.D.
13	1 (0.6)	N.D.	1 (1.1)	N.D.	N.D.	N.D.
14	3 (1.7)	1 (1.3)	2 (2.1)	N.D.	N.D.	N.D.
15	1 (0.6)	N.D.	1 (1.1)	N.D.	N.D.	N.D.
35	1 (0.6)	N.D.	1 (1.1)	N.D.	N.D.	N.D.

The frequency is shown as a percentage in parentheses.

The octamer unit that is the same as BK-SE36 is shown in bold.

N.D. not detected.

**Figure 5:**
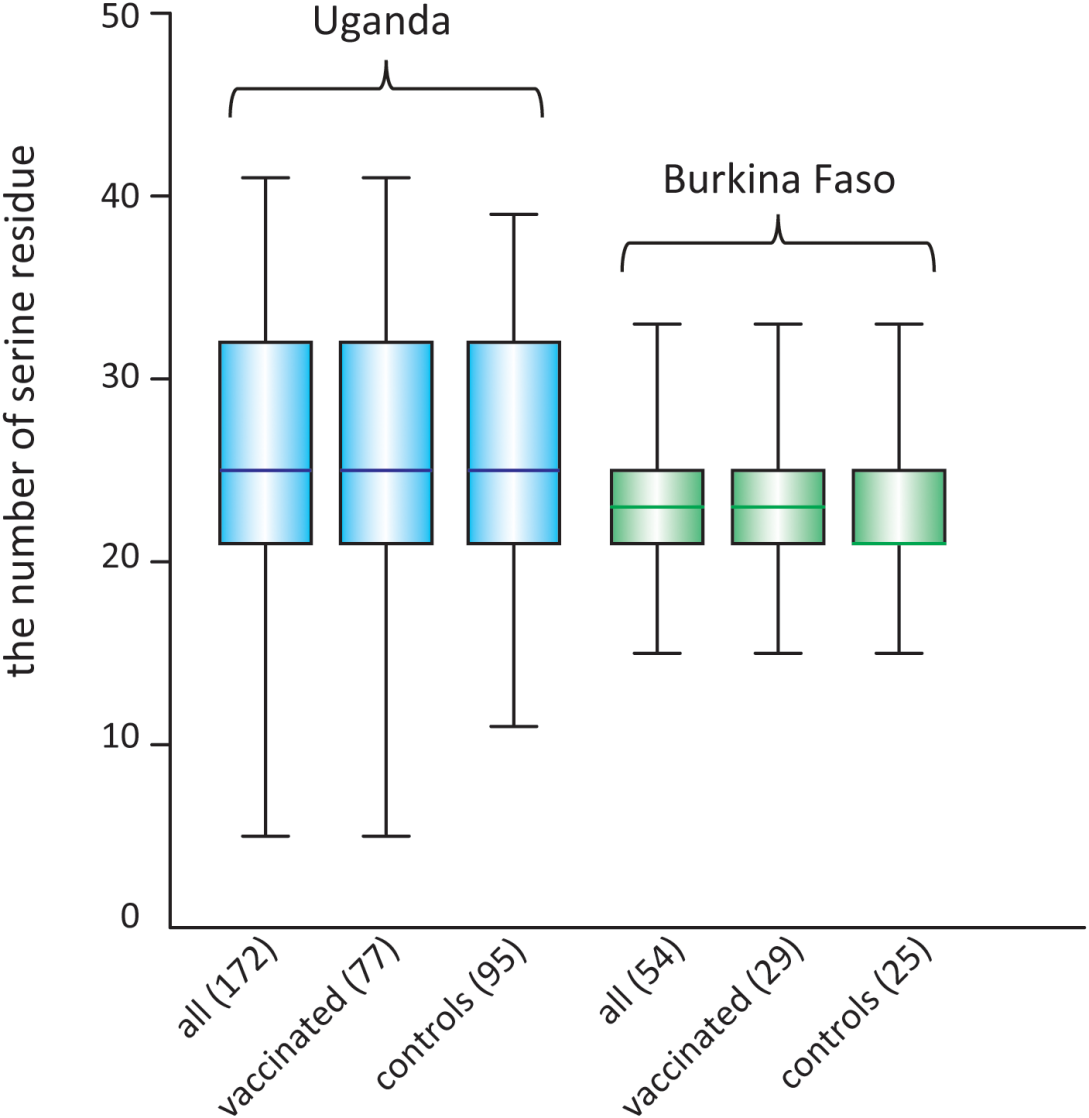
Box plot showing the number of poly-serine residues in the stretch of serine tandem repeats in the SR region of SERA5, in isolates from two clinical trial sites. The number of analyzed sequences is shown in parentheses.

For the serine/proline substitution in the 13-mer insertion/deletion part of the SR region, the frequency of proline substitution was not biased between the vaccinated and control groups in both the Uganda and Burkina Faso isolates (*p* > 0.05, χ2-test) (**Table 6**). For the 17-mer dimorphic region where the vaccine type is Type IV, the frequency of Type IV isolates did not greatly differ from those obtained from the BK-SE36 vaccine group and control subjects (*p* > 0.05, χ2-test) (**Table 6**). Considering the number of serine residues between the vaccinated and control groups (**Figure 5**, **Supplementary Table S6**), the variation in the length of the serine stretch also did not differ between the two treatment arms (BK-SE36 or control arms) (*p* > 0.05, Mann-Whitney test). However, it is noted that the number of serine residues was relatively greater (*i.e*. longer stretch of serine residues) in the Ugandan isolates than those isolated from Burkina Faso (*p* < 0.05, Mann-Whitney test).

**Table 6:** Frequency of proline in 13-mer insertion/deletion sequence and frequency of Type IV sequence in the 17-mer dimorphic sequence of the SERA5 SR region in Uganda and Burkina Faso.

	n	No. of Proline	frequency (%)	No. of Type IV	frequency (%)
Uganda + Burkina Faso	226	60	26.5	43	19.0
Vaccinated	106	29	27.4	21	19.8
Controls	120	31	25.8	22	18.3
Uganda	172	47	27.3	35	20.3
Vaccinated	77	21	27.3	16	20.8
Controls	95	26	27.4	19	20.0
Burkina Faso	54	13	24.1	8	14.8
Vaccinated	29	8	27.6	5	17.2
Controls	25	5	20.0	3	12.0

Genetic differentiation in the SE36 region of SERA5 was examined using *Fst*, the Wright’s fixation index of inter-population variance in allele frequencies (Wright, 1965) (**Table 7**). The *Fst* value suggested a significant difference in the SE36 region of SERA5 and SR region between the Ugandan and Burkina Faso isolates (*p* < 0.05). However, no significant differentiation was detected between isolates from two vaccination arms (BK-SE36 or control). The OR region did not significantly differ between the Uganda and Burkina Faso isolates.

**Table 7:** Genetic differentiation (*Fst*) of the vaccine-type SE36 in SERA5 among isolates from clinical trial sites.

	*Fst* (*p*-value)
***Fst* of SERA5 BK-SE36 region**	
Uganda vs Burkina Faso	0.0248 (0.000)
Uganda: vaccinated group vs control group	-0.0028 (0.613)
Burkina Faso: vaccinated group vs control group	-0.0017 (0.396)
***Fst* of SERA5 octamer repeat region**	
Uganda vs Burkina Faso	0.0073 (0.099)
Uganda: vaccinated group vs control group	-0.0032 (0.622)
Burkina Faso: vaccinated group vs control group	0.0040 (0.351)
***Fst* of SERA5 serine repeat region**	
Uganda vs Burkina Faso	0.0615 (0.000)
Uganda: vaccinated group vs control group	-0.0018 (0.414)
Burkina Faso: vaccinated group vs control group	-0.0172 (0.703)

## Discussion

Antigen polymorphisms driving allele-specific efficacy are a common limitation in malaria vaccine development (Thera et al., 2011; Ouattara et al., 2013; Neafsey et al., 2015). Early-stage clinical trials demonstrated that the BK-SE36 vaccine had acceptable reactogenicity, had no unexpected safety concerns, and was immunogenic. As a blood-stage vaccine, BK-SE36 is expected to reduce morbidity and mortality from *P. falciparum* infections. Indeed, in the longitudinal study following the clinical trial in Uganda, the BK-SE36 vaccine arm showed substantial differences in the hazard ratio to first episodes of high parasitemia + fever (0.28, 95% CI: 0.12-0.66, *p* < 0.01) and against all malaria episodes of high parasitemia + fever (0.34, 95% CI: 0.15-0.76, *p* = 0.01) (Palacpac et al., 2013). This promising efficacy could be due to the high frequency of identical/homologous alleles to vaccine-type SE36 at the clinical trial site, however, the current analysis indicates that the frequency of homologous alleles was very low, not only in Uganda (where promising data were obtained) but also in Burkina Faso as well as other African countries such as Tanzania and Ghana. When SERA5 sequences from the BK-SE36 vaccinated and control group were compared, the difference between the frequency of the identical or homologous allele to vaccine-type SE36 was within statistical error, showing no evidence of vaccination-induced allele selection. In addition, no selection of the SERA5 haplotype was evident in the BK-SE36 treatment arm.

SERA5 has markedly fewer SNPs than the malaria vaccine candidates AMA1 and CSP, although it is the recombination events that generated much of the diversity in the number of octamer repeats and number of serine residues in the serine stretch in SERA5. The consensus sequence of SERA5 was identical in the four African countries. The genetic differentiation in SERA5 (observed in SNPS and repeat variants found in the OR and SR regions) among isolates from Uganda and Burkina Faso may be due to geographical distance. This finding was similar to an earlier inference using worldwide isolates from 9 countries, albeit isolates from Africa were sourced only from 2 countries (Tanabe et al., 2012). The biased geographical distribution for SNPs and repeat variants may possibly be a result of gene flow barriers or divergent selection in different populations resulting in high-level sequence conservation of *sera5*, except for the insertion/deletion and repeat regions.

Both OR and SR regions, hot spots of recombination, are characterized as intrinsically unstructured (Yagi et al., 2014). The epitope mapping data suggest that (i) intrinsically unstructured regions allow some flexibility for the epitopes to interact with other molecules/proteins; (ii) the regions adjacent to the repeats are not strictly disordered as they showed a tendency to form a secondary structure; (iii) the protective epitopes of BK-SE36 are located in the N-terminal region where the repeat number of octamer units varied among alleles; and (iv) serum from mice and squirrel monkeys vaccinated with BK-SE36 also showed a broad range of reactivity against peptides covering the SR region (Yagi et al., 2014). Indeed, serum samples from clinical trial participants that received BK-SE36 preferentially recognized epitopes corresponding to the SR and flanking regions (including 13-mer insertion/deletion, polyserine residue and 17-mer dimorphic region) (Ezoe et al., 2020; Bougouma et al., 2022). Thus, these indicate that the antibodies induced by BK-SE36 vaccination bound, with reproducibility, to epitopes located in the disordered regions of the protein. Further studies with larger sample size, in combination with protective efficacy data, would fine-tune which epitopes would be immunologically important with respect to vaccine development.

Limited polymorphism of SERA5 could be attributed a to low seropositive population even in malaria-endemic regions. For example, a seroepidemiology study in the Solomon Islands showed that <50% of adults and <10% of children under 10 years were seropositive to anti-SE36, although higher seropositivity to MSP-1 was observed in the population (Horii et al., 2010). In the phase 1b BK-SE36 trial in Uganda, almost no seroconversion was evident in vaccinated adults (18-32 years old), but notably, around 70% of 6-10-year-old in the BK-SE36 arm were seropositive after two BK-SE36 vaccinations (i.e. seropositivity is defined as having ≥2-fold increase in anti-SE36 antibody titers compared to baseline) (Palacpac et al., 2013). The low seroconversion rate observed in adult subjects living in malaria-endemic areas is in contrast to an early-stage trial in malaria naïve Japanese adults where 100% seroconversion was achieved after two or three vaccinations of BK-SE36 (Horii et al., 2010). These data suggest that immune tolerance occurs in malaria-endemic areas through repeated infections. Recently, SE36 was demonstrated to tightly bind the host protein vitronectin and the resulting complex cloaks the merozoite surface allowing the parasite to circumvent host immunity (Tougan et al., 2018). We contend that the repeated presentation of vitronectin-bound-SE36, as a result of infection, was exploited by the parasite such that SE36 disguises itself as a host antigen, avoiding clearance by phagocytosis and leading to the gradual acquisition of immune tolerance. If this interpretation is true, young children or individuals with limited malaria infection history would respond better to BK-SE36 vaccination similar to malaria naïve Japanese adults. Indeed, the seroconversion rate was higher in the 24–60-month-old and 12–24-month-old Burkinabe children (79–83% after two- and 89–97% after three-vaccinations) (Bougouma et al., 2022). This immune tolerance could also explain why SERA5 is less likely to be under substantial immune selection pressure compared to other blood-stage malaria vaccine antigens such as AMA1 and CSP. Moving forward, an important caveat to our data interpretation would be how to overcome immune tolerance in hyporesponsive populations.

Overall, despite polymorphism and mismatches observed in SERA5 repeat regions and in some SNPs between Af-cons and vaccine-type SE36, BK-SE36 is a promising vaccine candidate, especially for infants and malaria naïve travelers. In the current analyses, samples with multiple *P. falciparum* infections were excluded, so a direct comparison of the number of infections is not possible based solely on the number of SERA5 sequences. In the Ugandan follow-up study, based on different parasite density thresholds, BK-SE36 may likely have a disease ameliorating effect rather than preventing infection per se (Palacpac et al., 2013). Differences were observed among isolates from the four African countries in the number of consecutive serine residues in the SR region and the frequency of Type I to IV sequences in the 17-mer dimorphic region. How these differences could affect the efficacy of the vaccine is not known. Larger efficacy proof-of-concept trials are needed.

## Data availability statement

The datasets presented in this study can be found in online repositories. The names of the repository/repositories and accession number(s) can be found in the article/**Supplementary Materials**.

## Ethics statement

The studies involving human 1: participants were reviewed and approved by for the Ugandan trial and follow-up study the ethical institutional review committees of Med Biotech Laboratories (Ref# IRB-00003990-MBL-BIOMEDICAL, IRB-00003995-MBL-BIOMEDICAL), Uganda National Council for Science and Technology (Ref# HS635, HS866), Osaka University (Ref# 20-3, 287), and Research Foundation for Microbial Diseases of Osaka University. 2: Approval for the Burkina Faso trial was obtained from Comité d’Éthique pour la Recherche en Santé du Burkina Faso (Ref# 2014-12-144), Comité Institutionnel de Bioéthique du INSP/CNRFP (previous name: CNRFP) (Ref# N°2014/071/MS/SG/CNRFP/CIB, N°2016/000008/MS/SG/CNRFP/CIB), Agence Nationale de Régulation Pharmaceutique (ANRP, previous name: Direction Générale de la Pharmacie, du Médicament et des Laboratoires [DGPML], Ref# N°2015:658/MS/CAB), Scientific Committee/Institutional Review Committee of the Research Institute for Microbial Diseases (Ref# 26), Osaka University (Ref# 574); and London School of Hygiene and Tropical Medicine Research Ethics Committee (Ref# 9175). Written informed consent to participate in this study was provided by the participants’ legal guardian/next of kin.

## Author contributions

Conceptualization, NA, NP and TH. methodology, validation and formal analysis, NA. investigation and resources, NA, AY, BB, EN, BK, TE, EB, AT, IN, AD, SS, SH, FD, OL, TH. data curation, NA, NP, SH, and SS. writing—original draft preparation, NA, NP, and TH. writing—review and editing, BK, EN, BB, EB, SH, FD. visualization, NA, NP, and TH. funding acquisition, NP, SH, OL, SS, TE and TH. All authors contributed to the article and approved the submitted version.

## References

[B1] Aoki S. Li J. Itagaki S. Okech B. A. Egwang T. G. Matsuoka H.. (2002). Serine repeat antigen (SERA5) is predominantly expressed among the SERA multigene family of *Plasmodium falciparum*, and the acquired antibody titers correlate with serum inhibition of the parasite growth. J. Biol. Chem. 277, 47533–47540. doi: 10.1074/jbc.M207145200 12244052

[B2] Arisue N. Palacpac N. M. Q. Tougan T. Horii T. (2020). Characteristic features of the SERA multigene family in the malaria parasite. Parasitol. Vectors. 13, 170. doi: 10.1186/s13071-020-04044-y PMC7132891 32252804

[B3] Bougouma E. C. Palacpac N. M. Q. Tiono A. B. Nebie I. Ouédraogo A. Houard S.. (2022). Safety and immunogenicity of BK-SE36 in a blinded, randomized, controlled, age de-escalating phase ib clinical trial in Burkinabe children. Front. Immunol. 13, 978591. doi: 10.3389/fimmu.2022.978591 36119062 PMC9471861

[B4] Debrabant A. Maes P. Delplace P. Dubremetz J. F. Tartar A. Camus D. (1992). Intramolecular mapping of *Plasmodium falciparum* p126 proteolytic fragments by n-terminal amino acid sequencing. Mol. Biochem. Parasitol. 53, 89–96. doi: 10.1016/0166-6851(92)90010-h 1501648

[B5] Excoffier L. Lischer H. E. (2010). Arlequin suite ver 3.5: a new series of programs to perform population genetics analyses under Linux and windows. Mol. Ecol. Resour. 10, 564–567. doi: 10.1111/j.1755-0998.2010.02847.x 21565059

[B6] Ezoe S. Palacpac N. M. Q. Tetsutani K. Yamamoto K. Okada K. Taira M.. (2020). First-in-human randomised trial and follow-up study of *Plasmodium falciparum* blood-stage malaria vaccine BK-SE36 with CpG-ODN(K3). Vaccine. 38, 7246–7257. doi: 10.1016/j.vaccine.2020.09.056 33012605

[B7] Fox B. A. Xing-Li P. Suzue K. Horii. T. Bzik D. J. (1997). *Plasmodium falciparum*: An epitope within a highly conserved region of the 47-kDa amino-terminal domain of the serine repeat antigen is a target of parasite-inhibitory antibodies. Exp. Parasitol. 85, 121–134. doi: 10.1006/expr.1996.4118 9030663

[B8] Horii T. Shirai H. Li J. Ishii K. J. Palacpac N. Q. Tougan T.. (2010). Evidences of protection against blood-stage infection of *Plasmodium falciparum* by the novel protein vaccine SE36. Parasitol. Int. 59, 380–386. doi: 10.1016/j.parint.2010.05.002 20493274

[B9] Kumar S. Stecher G. Li M. Knyaz C. Tamura K. (2018). MEGA X: Molecular evolutionary genetics analysis across computing platforms. Mol. Biol. Evol. 35, 1547–1549. doi: 10.1093/molbev/msy096 29722887 PMC5967553

[B10] Laurens M. B. (2020). RTS,S/AS01 vaccine (Mosquirix™): An overview. Hum. Vaccin. Immunother. 16, 480–489. doi: 10.1080/21645515.2019.1669415 31545128 PMC7227679

[B11] Librado P. Rozas J. (2009). DnaSP v5: a software for comprehensive analysis of DNA polymorphism data. Bioinformatics. 25, 1451–1452. doi: 10.1093/bioinformatics/btp187 19346325

[B12] Li J. Matsuoka H. Mitamura T. Horii T. (2002). Characterization of proteases involved in the processing of *Plasmodium falciparum* serine repeat antigen (SERA). Mol. Biochem. Parasitol. 120, 177–186. doi: 10.1016/s0166-6851(01)00452-2 11897123

[B13] Maxmen A. (2021). Scientists hail historic malaria vaccine approval - but point to challenges ahead. Nature. doi: 10.1038/d41586-021-02755-5 34625728

[B14] Morimatsu K. Morikawa T. Tanabe K. Bzik D. J. Horii T. (1997). Sequence diversity in the amino-terminal 47 kDa fragment of the *Plasmodium falciparum* serine repeat antigen. Mol. Biochem. Parasitol. 86, 249–254. doi: 10.1016/s0166-6851(97)00038-8 9200130

[B15] Neafsey D. E. Juraska M. Bedford T. Benkeser D. Valim C. Griggs A.. (2015). Genetic diversity and protective efficacy of RTS,S/AS01 malaria vaccine. N. Engl. J. Med. 373, 2025–2037. doi: 10.1056/NEJMoa1505819 26488565 PMC4762279

[B16] Nei M. Gojobori T. (1986). Simple methods for estimating the numbers of synonymous and nonsynonymous nucleotide substitutions. Mol. Biol. Evol. 3, 418–426. doi: 10.1093/oxfordjournals.molbev.a040410 3444411

[B17] Ogutu B. R. Apollo O. J. McKinney D. Okoth W. Siangla J. Dubovsky F.. (2009). Blood stage malaria vaccine eliciting high antigen-specific antibody concentrations confers no protection to young children in Western Kenya. PloS One 4, e4708. doi: 10.1371/journal.pone.0004708 19262754 PMC2650803

[B18] Okech B. Mujuzi G. Ogwal A. Shirai H. Horii T. Egwang T. G. (2006). High titers of IgG antibodies against *Plasmodium falciparum* serine repeat antigen 5 (SERA5) are associated with protection against severe malaria in Ugandan children. Am. J. Trop. Med. Hyg. 74, 191–197. doi: 10.4269/ajtmh.2006.74.191 16474069

[B19] Okech B. A. Nalunkuma A. Okello D. Pang X. L. Suzue K. Li J.. (2001). Natural human immunoglobulin G subclass responses to *Plasmodium falciparum* serine repeat antigen in Uganda. Am. J. Trop. Med. Hyg. 65, 912–917. doi: 10.4269/ajtmh.2001.65.912 11791998

[B20] Ouattara A. Takala-Harrison S. Thera M. A. Coulibaly D. Niangaly A. Saye R.. (2013). Molecular basis of allele-specific efficacy of a blood-stage malaria vaccine: vaccine development implications. J. Infect. Dis. 207, 511–519. doi: 10.1093/infdis/jis709 23204168 PMC3537449

[B21] Owalla T. J. Palacpac N. M. Shirai H. Horii T. Egwang T. G. (2013). Association of naturally acquired IgG antibodies against *Plasmodium falciparum* serine repeat antigen-5 with reduced placental parasitemia and normal birth weight in pregnant Ugandan women: a pilot study. Parasitol. Int. 62, 237–239. doi: 10.1016/j.parint.2013.01.006 23395684

[B22] Palacpac N. M. Arisue N. Tougan T. Ishii K. J. Horii T. (2011). *Plasmodium falciparum* serine repeat antigen 5 (SE36) as a malaria vaccine candidate. Vaccine. 29, 5837–5845. doi: 10.1016/j.vaccine.2011.06.052 21718740

[B23] Palacpac N. M. Q. Horii T. (2020). Malaria vaccines: Facing unknowns. F1000Res. 9, F1000 Faculty Rev–296. doi: 10.12688/f1000research.22143.1 PMC7194457 32399189

[B24] Palacpac N. M. Ntege E. Yeka A. Balikagala B. Suzuki N. Shirai H.. (2013). Phase 1b randomized trial and follow-up study in Uganda of the blood-stage malaria vaccine candidate BK-SE36. PloS One 8, e64073. doi: 10.1371/journal.pone.0064073 23724021 PMC3665850

[B25] Pang X. L. Horii T. (1998). Complement-mediated killing of *Plasmodium falciparum* erythrocytic schizont with antibodies to the recombinant serine repeat antigen (SERA). Vaccine. 16, 1299–1305. doi: 10.1016/s0264-410x(98)00057-7 9682394

[B26] Pang X. L. Mitamura T. Horii T. (1999). Antibodies reactive with the N-terminal domain of *Plasmodium falciparum* serine repeat antigen inhibit cell proliferation by agglutinating merozoites and schizonts. Infect. Immun. 67, 1821–1827. doi: 10.1128/IAI.67.4.1821-1827.1999 10085023 PMC96533

[B27] Proietti C. Pettinato D. D. Kanoi B. N. Ntege E. Crisanti A. Riley E. M. (2011). Continuing intense malaria transmission in northern Uganda. Am. J. Trop. Med. Hyg. 84, 830–837. doi: 10.4269/ajtmh.2011.10-0498 21540398 PMC3083756

[B28] Stallmach R. Kavishwar M. Withers-Martinez C. Hackett F. Collins C. R. Howell S. A.. (2015). *Plasmodium falciparum* SERA5 plays a non-enzymatic role in the malarial asexual blood-stage lifecycle. Mol. Microbiol. 96, 368–387. doi: 10.1111/mmi.12941 25599609 PMC4671257

[B29] Sugiyama T. Suzue K. Okamoto M. Inselburg J. Tai. K. Horii T. (1996). Production of recombinant SERA proteins of *Plasmodium falciparum* in *Escherichia coli* by using synthetic genes. Vaccine. 14, 1069–1076. doi: 10.1016/0264-410x(95)00238-v 8879104

[B30] Takala S. L. Coulibaly D. Thera M. A. Batchelor A. H. Cummings M. P. Escalante A. A.. (2009). Extreme polymorphism in a vaccine antigen and risk of clinical malaria: implications for vaccine development. Sci. Transl. Med. 1, 2ra5. doi: 10.1126/scitranslmed.3000257 PMC2822345 20165550

[B31] Tanabe K. Arisue N. Palacpac N. M. Yagi M. Tougan T. Honma H.. (2012). Geographic differentiation of polymorphism in the *Plasmodium falciparum* malaria vaccine candidate gene SERA5. Vaccine. 30, 1583–1593. doi: 10.1016/j.vaccine.2011.12.124 22230587

[B32] Tanabe K. Mita T. Jombart T. Eriksson A. Horibe S. Palacpac N.. (2010). *Plasmodium falciparum* accompanied the human expansion out of Africa. Curr. Biol. 20, 1283–1289. doi: 10.1016/j.cub.2010.05.053 20656209

[B33] Thera M. A. Doumbo O. K. Coulibaly D. Laurens M. B. Ouattara A. Kone A. K.. (2011). A field trial to assess a blood-stage malaria vaccine. N. Engl. J. Med. 365, 1004–1013. doi: 10.1056/NEJMoa1008115 21916638 PMC3242358

[B34] Tiono A. B. Kangoye D. T. Rehman A. M. Kargougou D. G. Kaboré Y. Diarra A.. (2014). Malaria incidence in children in south-West Burkina Faso: comparison of active and passive case detection methods. PloS One 9, e86936. doi: 10.1371/journal.pone.0086936 24475198 PMC3901722

[B35] Tougan T. Edula J. R. Takashima E. Morita M. Shinohara M. Shinohara A.. (2018). Molecular camouflage of *Plasmodium falciparum* merozoites by binding of host vitronectin to P47 fragment of SERA5. Sci. Rep. 8, 5052. doi: 10.1038/s41598-018-23194-9 29567995 PMC5864917

[B36] Weiss D. J. Lucas T. C. D. Nguyen M. Nandi A. K. Bisanzio D. Battle K. E.. (2019). Mapping the global prevalence, incidence, and mortality of *Plasmodium falciparum*, 2000-17: A spatial and temporal modelling study. Lancet, 394, 322–331. doi: 10.1016/S0140-6736(19)31097-9 31229234 PMC6675740

[B37] WHO (2021) World malaria report. Available at: https://www.who.int/teams/global-malaria-programme/reports/world-malaria-report-2021 (Accessed August 15, 2022).

[B38] Wright S. (1965). The interpretation of population structure by F-statistics with special regard to systems of mating. Evolution. 19, 395–420. doi: 10.1111/j.1558-5646.1965.tb01731.x

[B39] Yagi M. Bang G. Tougan T. Palacpac N. M. Arisue N. Aoshi T.. (2014). Protective epitopes of the *Plasmodium falciparum* SERA5 malaria vaccine reside in intrinsically unstructured n-terminal repetitive sequences. PloS One 9, e98460. doi: 10.1371/journal.pone.0098460 24886718 PMC4041889

[B40] Yagi M. Palacpac N. M. Ito K. Oishi Y. Itagaki S. Balikagala B.. (2016). Antibody titres and boosting after natural malaria infection in BK-SE36 vaccine responders during a follow-up study in Uganda. Sci. Rep. 6, 34363. doi: 10.1038/srep34363 27703240 PMC5050508

[B41] Yeoh S. O’Donnell R. A. Koussis K. Dluzewski A. R. Ansell K. H. Osborne S. A.. (2007). Subcellular discharge of a serine protease mediates release of invasive malaria parasites from host erythrocytes. Cell. 131, 1072–1083. doi: 10.1016/j.cell.2007.10.049 18083098

