# Supplementary material for: African-specific polymorphisms in Plasmodium falciparum serine repeat antigen 5 in Uganda and Burkina Faso clinical samples do not interfere with antibody response to BK-SE36 vaccination
Source: Front Cell Infect Microbiol. 2022 Dec 16;12:1058081. doi: 10.3389/fcimb.2022.1058081 (PMC9802637; doi:10.3389/fcimb.2022.1058081)
Supplement: Supplementary file 1 [file DataSheet_1.pdf]

Supplementary Material

Supplementary Figure S1 Polymorphism in SERA5 octamer repeat (OR) region from 4 African countries. OR is classified into two groups; group I consisted of six subgroups (Ia–If) and group II. Main motifs in subgroups Ia, Ib, Ic, Id, Ie, and If were TGESQTGN, TGGGQAGN, TVGDQAGS, TGGSPQGS, TGASPQGS, and TGASQPGS and the motif in group II was SEPSNPVS. Amino acid substitutions are shown in red. Haplotypes No. 1–36 were previously reported in Tanabe et al. (2012). Haplotypes No. 37–80 were newly numbered in this study. Haplotypes identical to representative laboratory strains are shown in parentheses.identical with representative laboratory strains are shown in parentheses.

No. of Haplotype	4 countries total	Uganda			Burkina Faso			Tanzania	Ghana	Ia			Ib				Ic	Id		Ie		If	II						
		total	vaccinated	controls	total	vaccinated	controls																						
36	1							1										TGGSPQGS		TGASPQGS	x11 unit	TGASQPGS	SEPSNPVS						
1	2							1	1	TGES												QPGS	SEPSNPVS						
37	1	1		1						TGES							QAGS	TGGSPQGS		TGASPQGS	x3 unit	TGASQPGS	SEPSNPVS						
38	1	1	1							TGESQTGN			TGGGQAGN					TGGSPQGS				TGASQPGS	SEPSNPVS						
31	1							1		TGESQTGN			TGGGQAGN				TVGDQAGS	TG		ASPQGS TGASPQGS	x7 unit	TGASQPGS	SEPSNPVS						
2(Honduras-1)	5	4	3	1					1	TGESQTGN			TGGGQAGN				TVGDQAGS	TGGSPQGS				TGASQPGS	SEPSNPVS						
39	1				1		1			TGESQTGN			AGGGQAGN				TVGDQAGS	TGGSPQGS				TGASQPGS	SEPSNPVS						
40	2	2		2						TGESQTGN			TVGD QAGN				TVGDQAGS	TGGSPQGS				TGASQPGS	SEPSNPVS						
41	1	1		1						TGESQTGN			TGGGQAGN				TVGDQAGS	TGGSPQGS	TGGSPQGS			TGASQPGS	SEPSNPVS						
42	1				1	1				TGESQTGN			TGGGQAGN				TVGDQAGS	TGGSPQGS		TGASPQGS		TGASQPGS							
3	8	7	3	4				1		TGESQTGN			TGGGQAGN				TVGDQAGS	TGGSPQGS		TGASPQGS		TGASQPGS	SEPSNPVS						
43	9	9	4	5						TGESQTRN			TGGGQAGN				TVGDQAGS	TGGSPQGS		TGASPQGS		TGASQPGS	SEPSNPVS						
4	1								1	TGESQTGN			TGGGQAGN				TVGDQAGS	TGRSPQGS		TGASPQGS		TGASQPGS	SEPSNPVS						
5	9	6	2	4	1		1	1	1	TGESQTGN			TGGGQAGN				TVGDQAGS	TGGSPQGS		TGASPQGS	x2 unit	TGASQPGS	SEPSNPVS						
6	8	2	1	1	2	1	1	4		TGESQTGN			TGGGQAGN				TVGDQAGS	TGGSPQGS		TGASPQGS	x3 unit	TGASQPGS	SEPSNPVS						
7	4	2	1	1	1	1			1	TGESQTGN			TGGGQAGN				TVGDQAGS	TGGSPQGS		TGASPQGS	x4 unit	TGASQPGS	SEPSNPVS						
8	5	2	1	1	1	1		2		TGESQTGN			TGGGQAGN				TVGDQAGS	TGGSPQGS		TGASPQGS	x5 unit	TGASQPGS	SEPSNPVS						
44	3	3	1	2						TGESQTGN			TGGGQAGN				TVGDQAGS	TGGSPQGS		TGASPQGS	x6 unit	TGASQPGS	SEPSNPVS						
45	1	1		1						TGGG QTGN			TGGGQAGN				TVGDQAGS	TGGSPQGS		TGASPQGS	x6 unit	TGASQPGS	SEPSNPVS						
9	3	1		1				2		TGESQTGN			TGGGQAGN				TVGDQAGS	TGGSPQGS		TGASPQGS	x7 unit	TGASQPGS	SEPSNPVS						
46	3	3	1	2						TGESQTGN			TGGGQAGN				TVGDQAGS	TGGSPQGS		TGASPQGS	x8 unit	TGASQPGS	SEPSNPVS						
47	1	1		1						TGESQTGN			TGGGQAGN				TVGDQAGS	TGGSPQGS		TGASPQGS	x9 unit	TGASQPGS	SEPSNPVS						
48	1	1		1						TGESQTGN			TGGGQAGN				TVGDQAGS	TGGSPQGS		TGASPQGS	x29 unit	TGASQPGS	SEPSNPVS						
49	1	1		1						TGESQTGN			TGGGQAGN				TVGDQAGS	TGGSPQGS				TGASQPGS	SEPSNPVS	SEPSNPVS	SEPSNPVS				
50	2	1	1		1	1				TGESQTGN			TGGGQAGN				TVGDQAGS	TGGSPQGS				TGASQPGS	SEPSNPVS	SEPSNPVS	SEPSSPVS	SEPSNPVS			
51	1	1	1							TGESQTGN			TGGGQAGN	TGG			DQAGS			TGASPQGS	x7 unit	TGASQPGS	SEPSNPVS						
24	2				1		1	1		TGESQTGN			TGGGQAGN	TGG			DQAGS	TGGSPQGS				TGASQPGS	SEPSNPVS						
72	1				1	1				TGESQTGN			TGGGQAGN	TGG			DQAGS	TGGSPQGS				TGASQPGS	SEPSNPVS	SEPSNPVS	SEPSNPVS	SEPSNPVS			
52	2	1	1		1	1				TGESQTGN			TGGGQAGN	TGG			DQAGS	TGGSPQGS		TGASPQGS		TGASQPGS	SEPSNPVS						
80(3D7)	4	2	1	1	2	2				TGESQTGN			TGGGQAGN	TGG			DQAGS	TGGSPQGS		TGASPQGS	x2 unit	TGASQPGS	SEPSNPVS						
53	1	1	1							TGGG QTGN			TGGGQAGN	TGG			DQAGS	TGGSPQGS		TGASPQGS	x2 unit	TGASQPGS	SEPSNPVS						
54	1	1	1							TGESQTGN			TGGGQAGN	TGG			DQAGS	TGGSPQGS		TGASPQGS	x3unit	TGASQPGS	SEPSNPVS						
25	2				1	1			1	TGESQTGN			TGGGQAGN	TGG			DQAGS	TGGSPQGS		TGASPQGS	x4 unit	TGASQPGS	SEPSNPVS						
55	1	1		1						TGESQTGN			TGGGQAGN	TGG			DQAGS	TGGSPQGS		TGASPQGS	x5 unit	TGASQPGS	SEPSNPVS						
26	1								1	TGESQTGN			TGGGQAGN	TGG			DQAGS	TGGSPQGS		TGASPQGS	x6 unit	TGASQPGS	SEPSNPVS						
56	1	1		1						TGGG QTGN			TGGGQAGN	TGG			DQAGS	TGGSPQGS		TGASPQGS	x5unit	TGASQPGS	SEPSNPVS						
57	1	1		1						TGESQTGN			TGGGQAGN	TGGG			QAGS	TGGSPQGS				TGASQPGS	SEPSNPVS						
28	2							2		TGESQTGN			TGGGQAGN	TGGG			QAGS	TGGSPQGS				TGASQPGS	SEPSNPVS	SEPSNPVS					
58	1				1		1			TGESQTGN			TGGGQAGN	TGGG			QAGS	TGGSPQGS		TGASPQGS	x4 unit	TGASQPGS	SEPSNPVS	SEPSNPVS					
16	1								1	TGESQTGN			TGGGQAGN	TVGD QAGN			TVG	SPQGS		TGASPQGS		TGASQPGS	SEPSNPVS						
17	2	1	1					1		TGESQTGN			TGGGQAGN	TVGD QAGN			TVG	SPQGS		TGASPQGS	x2 unit	TGASQPGS	SEPSNPVS						
59	1				1		1			TGESQTGN			TGGGQAGN	TVGD QAGN			TVG	SPQGS		TGASPQGS	x5 unit	TGASQPGS	SEPSNPVS						
18	1								1	TGESQTGN			TGGGQAGN	TVGD QAGN			TVG	SPQGS		TGASPQGS	x9 unit	TGASQPGS	SEPSNPVS						
60	1				1	1				TGESQTGN			TGGGQAGN	TGGGQAGN			TVGDQAGS					TGASQPGS	SEPSNPVS						
61	1				1		1			TGESQTGN			TGGGQAGN	TGGGQAGN			TVGDQAGS			TGASPQGS		TGASQPGS	SEPSNPVS						
62	1	1		1						TGESQTGN			TGGGQAGN	TGGGQAGN			TVGDQAGS	TGGS				QPGS	SEPSNPVS	SEPSNPVS					
10(FCR3)	103	55	24	31	13	5	8	24	11	TGESQTGN			TGGGQAGN	TGGGQAGN			TVGDQAGS	TGGSPQGS				TGASQPGS	SEPSNPVS						
11	2	1	1					1		TGESQTRN			TGGGQAGN	TGGGQAGN			TVGDQAGS	TGGSPQGS				TGASQPGS	SEPSNPVS						
63	1	1		1						TGESQTGN			TGGGQAGN	TGVGQAGN			TVGDQAGS	TGGSPQGS				TGASQPGS	SEPSNPVS						
64	1	1	1							TGESQTGN			TGGGQAGN	TGGDQAGN			TVGDQAGS	TGGSPQGS				TGASQPGS	SEPSNPVS						
65	1				1		1			TGESQTGN			TGGGQAGN	TGGGQAAN			TVGDQAGS	TGGSPQGS				TGASQPGS	SEPSNPVS						
66	1				1		1			TGESQTGN			TGGDQAGN	TGGGQAGN			TVGDQAGS	TGGSPQGS				TGASQPGS	SEPSNPVS						
67	1	1		1						TGESQTGN			TGGDQADN	TGGGQAGN			TVGDQAGS	TGGSPQGS				TGASQPGS	SEPSNPVS						
15	18	14	8	6	1		1	3		TGESQTGN			TGGGQAGN	TVGD QAGN			TVGDQAGS	TGGSPQGS				TGASQPGS	SEPSNPVS						
20	17	9	7	2	3		3	2	3	TGESQTRN			TGGGQAGN	TVGD QAGN			TVGDQAGS	TGGSPQGS				TGASQPGS	SEPSNPVS						
19	10	8	3	5	1	1		1		TGESQTGN			AGGGQAGN	TVGD QAGN			TVGDQAGS	TGGSPQGS				TGASQPGS	SEPSNPVS						
68	2	1	1		1	1				TGESQTGN			TGGGQAGN	TGGGQAGN			TVGDQAGS	TGGSPQGS				TGASQPGS	SEPSNPVS	SEPSNPVS					
29	4	2		2	1		1		1	TGESQTGN			TAGGQAGN	TAGGQAGN			TVGDQAGS	TGGSPQGS				TGASQPGS	SEPSNPVS	SEPSNPVS					
69	2	2	1	1						TGESQTGN			TGGGQAGN	TGGGQAGN			TVGDQAGS	TGGSPQGS				TGASQPGS	SEPSNPVS	SEPSNPVS	SEPSNPVS				
70	2	2		2						TGESQTGN			TAGGQAGN	TAGGQAGN			TVGDQAGS	TGGSPQGS				TGASQPGS	SEPSNPVS	SEPSNPVS	SEPSNPVS				
71	1				1	1				TGESQTGN			TAGGQAGN	TAGGQAGN			TVGDQAGS	TGGSPQGS				TGASQPGS	SEPSNPVS	SEPSNPVS	SEPSNPVS	SEPSNPVS	SEPSNPVS		
30	1								1	TGESQTGN			TAGGQAGN	TAGGQAGN			TVGDQAGS	TGGSPQGS				TGASQPGS	SEPSNPVS	SEPSNPVS	SEPSNPVS	SEPSNPVS	SEPSNPVS	SEPSNPVS	
13	9	8	3	5				1		TGESQTGN			TGGGQAGN	TGGGQAGN	TGGGQAGN		TVG	SPQGS				TGASQPGS	SEPSNPVS						
12	8	1	1		2	1	1	2	3	TGESQTGN			TGGGQAGN	TGGGQAGN	TGGGQAGN		TVGDQAGS	TGGSPQGS				TGASQPGS	SEPSNPVS						
35	1								1	TGGG QTGN			TGGGQAGN	TGGGQAGN	TGGGQAGN		TVGDQAGS	TGGSPQGS				TGASQPGS	SEPSNPVS						
73	1	1	1							TGESQTGN			TGGGQAGN	TVGD QAGN	TVGD QAGN		TVGDQAGS	TGGSPQGS				TGASQPGS	SEPSNPVS						
21	11	1		1	6	5	1		4	TGESQTRN			TGGGQAGN	TVGD QAGN	TVGD QAGN		TVGDQAGS	TGGSPQGS				TGASQPGS	SEPSNPVS						
74	1				1	1				TGESQTGN			TGGGQAGN	TGGGQAGN	TGGGQAGN		TVGDQAGS	TGGSPQGS				TGASQPGS	SVPSNPVS						
75	1				1	1				TGESQTRN			TGGGQAGN	TVG DQAGN	TGGGQAGN		TVGDQAGS	TGGSPQGS				TGASQPGS	SEPSNPVS						
76	1				1	1				TGESQTGN			TGGGQAGN	TVGD QAGN	TVGD QAGN	TGG	DQAGS	TGGSPQGS				TGASQPGS	SEPSNPVS						
77	2	2		2						TGESQTGN			TGGGQAGN	TGGGQAGN	TGGGQAGN	TGGGQAGN	TGV	SPQGS				TGASQPGS	SEPSNPVS						
79	1				1		1			TGESQTGN	TGESQTGN		TGGGQAGN				TGV	SPQGS				TGASQPGS	SEPSNPVS						
32	3				1	1		2		TGESQTGN	TGESQTGN		TGGGQAGN				TVGDQAGS	TGGSPQGS				TGASQPGS	SEPSNPVS						
33	2	1		1				1		TGESQTGN	TGESQTGN		TGGGQAGN				TVGDQAGS	TGGSPQGS				TGAIQPGS	SEPSNPVS						
34	1							1		TGESQTGN	TGGG QTGN		TGGGQAGN				TVGDQAGS	TGGSPQGS				TGASQPGS	SEPSNPVS	SEPSNPVS					
78	1	1	1							TGESQTGN	TGGG QTGN	TGGG QTGN	TGGGQAGN	TVGD QAGN			TVGDQAGS	TGGSPQGS				TGASQPGS	SEPSNPVS						
total	314	172	77	95	54	29	25	55	33																				

Supplementary Figure S2 Sequence variation in the SERA5 serine repeat (SR) region. Three components in the SR region were sequenced: the 13-mer insertion/deletion region, stretch of serine tandem repeats, and 17-mer dimorphic. Amino acid substitutions are shown in color. Haplotypes No. 1–56 were previously reported in Tanabe et al. (2012). Haplotypes No. 68–93 were newly numbered in this study. Haplotypes identical to representative laboratory strains are shown in parentheses.

No. of Haplotype	13 mer ins/del	Serine stretch	17mer dimorphic	4 countries total	Uganda total	Uganda vaccinated	Uganda controls	Burkina Faso total	Burkina Faso vaccinated	Burkina Faso controls	Tanzania	Ghana
1	TGTVRGDTESISD	SSSSS--------------------------------------	VNPPANGAGSTPDAKKK	3	1	1					1	1
3	TGTVRGDTESISD	SSSSSSSSSSSSSSS----------------------------	VNPPANGAGSTPDAKKK	7	2	1	1	2	1	1	2	1
68	TGTVRGDTESISD	SSSSSSSSSSSSSSSSS--------------------------	VNPPANGAGSTPDAKKK	1	1		1					
69	TGTVRGDTESISD	SSSSSSSSSSSSSSSSSSS------------------------	VNPPANGAGSTPDAKKK	2	1	1		1		1		
5	TGTVRGDTESISD	SSSSSSSSSSSSSSSSSSSSS----------------------	VNPPANGAGSTPDAKKK	53	27	8	19	15	7	8	6	5
6	TGTVRGDTEPISD	SISSSSSSSSSSSSSSSSSSS----------------------	VNPPANGAGSTPDAKKK	1							1	
7(K1)	TGTVRGDTEPISD	SSSSSSSSSSSSSSSSSSSSS----------------------	VNPPANGAGSTPDAKKK	17	9	4	5	6	4	2		2
8	TGTVRGDTESISD	SSSSSSSSSSSSSSSSSSSSSSS--------------------	VNPPANGAGSTPDAKKK	21	11	5	6	6	2	4	3	1
9	TGTVRGDTEPISD	SSSSSSSSSSSSSSSSSSSSSSS--------------------	VNPPANGAGSTPDAKKK	11	4		4	3	2	1	2	2
10	TGTVRGDTESISD	SSSSSSSSSSSSSSSSSSSSSSSSS------------------	VNPPANGAGSTPDAKKK	14	4	2	2	6	5	1	2	2
70	TGTVRGDTEPISD	SSSSSSSSSSSSSSSSSSSSSSSSS------------------	VNPPANGAGSTPDAKKK	3	3	2	1					
11	TGTVRGDTESISD	SSSSSSSSSSSSSSSSSSSSSSSSSSS----------------	VNPPANGAGSTPDAKKK	6	3	1	2	1		1	2	
12	TGTVRGDTESISD	SSSSSSSSSSSSSSSSSSSSSSSSSSSSS--------------	VNPPANGAGSTPDAKKK	2	1		1					1
13	TGTVRGDTESISD	SSSSSSSSSSSSSSSSSSSSSSSSSSSSSSS------------	VNPPANGAGSTPDAKKK	19	13	7	6	3	1	2	2	1
14	TGTVRGDTEPISD	SSSSSSSSSSSSSSSSSSSSSSSSSSSSSSS------------	VNPPANGAGSTPDAKKK	3	2	1	1					1
15	TGTVRGDTESISD	SRSSSSSSSSSSSSSSSSSSSSSSSSSSSSSSS----------	VNPPANGAGSTPDAKKK	1								1
16	TGTVRGDTESISD	SSSSSSSSSSSSSSSSSSSSSSSSSSSSSSSSS----------	VNPPANGAGSTPDAKKK	8	5	3	2	2	2			1
71	TGTVRGDTEPISD	SSSSSSSSSSSSSSSSSSSSSSSSSSSSSSSSS----------	VNPPANGAGSTPDAKKK	1				1		1		
72	TGTVRGDTESISD	SSSSSSSSSSSSSSSSSSSSSSSSSSSSSSSSSSS--------	VNPPANGAGSTPDAKKK	2	2	1	1					
73	TGTVRGDTESISD	SSSSSSSSSSSSSSSSSSSSSSSSSSSSSSSSSSSSS------	VNPPANGAGSTPDAKKK	1	1	1						
17	TGTVRGDTESISD	SSSSSSSSSSSSSSSSSSSSSSSSSSSSSSSSSSSSSSSSS--	VNPPANGAGSTPDAKKK	1								1
18	TGTVRGDTESISD	SSSSSSSSSSSSSSSSSSSSSSSSSSSSSSSSSSSSSSSSSSS	VNPPANGAGSTPDAKKK	1								1
19	TGTVRGDTESISD	SSSSSSSSSSSSSSS----------------------------	VNPPANGAGSTPDAKKR	4	3	3					1	
20	TGTVRGDTESISD	SSSSSSSSSSSSSSSSSSSSS----------------------	VNPPANGAGSTPDAKKR	2							1	1
21	TGTVRGDTEPISD	SSSSSSSSSSSSSSSSSSSSS----------------------	VNPPANGAGSTPDAKKR	4	1		1				3	
74	TGTVRGDTESISD	SSSSSSSSSSSSSSSSSSSSSSS--------------------	VNPPANGAGSTPDAKKR	1	1	1						
22	TGTVRGDTEPISD	SSSSSSSSSSSSSSSSSSSSSSSSS------------------	VNPPANGAGSTPDAKKR	2	1	1					1	
23	TGTVRGDTESISD	SSSSSSSSSSSSSSSSSSSSSSSSSSSSSSS------------	VNPPANGAGSTPDAKKR	3	1		1				2	
24	TGTVRGDTEPISD	SSSSSSSSSSSSSSSSSSSSSSSSSSSSSSS------------	VNPPANGAGSTPDAKKR	3	2	1	1				1	
75	TGTVRGDTEPISD	SSSSSSSSSSSSSSSSSSSSSSSSSSSSSSSSSSSSSSSSS--	VNPPANGAGSTPDAKKR	1	1	1						
25	TGTVRGDTESISD	SSSSSSSSSSS--------------------------------	VNPPANGPDSPTVKPPR	1							1	
76	TGTVRGDTESISD	SSSSSSSSSSSSSSS----------------------------	VNPPANGPDSPTVKPPR	2	2	2						
77	TGTVRGDTESISD	SSSSSSSSSSSSSSSSS--------------------------	VNPPANGPDSPTVKPPR	2	2		2					
78	TGTVRGDTESISD	SSSSSSSSSSSSSSSSSSSSS----------------------	VNPPANGPDSPTVKPPR	3	3	1	2					
26	TGTVRGDTEPISD	SSSSSSSSSSSSSSSSSSSSS----------------------	VNPPANGPDSPTVKPPR	6	2	2					4	
79	TGTVRGDTESISD	SSSSSSSSSSSSSSSSSSSSSSSSS------------------	VNPPANGPDSPTVKPPR	1	1		1					
27	TGTVRGDTEPISD	SSSSSSSSSSSSSSSSSSSSSSSSSSSSS--------------	VNPPANGPDSPTVKPPR	2	1		1				1	
28	TGTVRGDTESISD	SSSSSSSSSSSSSSSSSSSSSSSSSSSSSSS------------	VNPPANGPDSPTVKPPR	30	25	11	14				5	
29	TGTVRGDTEPISD	SSSSSSSSSSSSSSSSSSSSSSSSSSSSSSS------------	VNPPANGPDSPTVKPPR	1								1
80	TGTVRGDTESISD	SSSSSSSSSSSSSSSSSSSSSSSSSSSSSSSSSSSSSSS----	VNPPANGPDSPTVKPPR	1	1		1					
81	TGTVRGDTEPISD	SSSSSSSSSSS--------------------------------	ESLPANGPDSPTVKPPR	1	1		1					
30	TGTVRGDTEPISD	SSSSSSSSSSSSSSS----------------------------	ESLPANGPDSPTVKPPR	4							3	1
82	TGTVRGDTESISD	SSSSSSSSSSSSSSS----------------------------	ESLPANGPDSPTVKPPR	1				1		1		
32	TGTVRGDTESISD	SSSSSSSSSSSSSSSSSSS------------------------	ESLPANGPDSPTVKPPR	1							1	
33	TGTVRGDTEPISD	SSSSSSSSSSSSSSSSSSSSS----------------------	ESLPANGPDSPTVKPPR	2	1		1				1	
83	TGTVRGDTESISD	SSSSSSSSSSSSSSSSSSSSS----------------------	ESLPANGPDSPTVKPPR	1	1		1					
34	TGTVRGDTESISD	SSSSSSSSSSSSSSSSSSSSSSS--------------------	ESLPANGPDSPTVKPPR	3	2	1	1				1	
84	TGTVRGDTEPISD	SSSSSSSSSSSSSSSSSSSSSSS--------------------	ESLPANGPDSPTVKPPR	4	3	2	1	1	1			
35	TGTVRGDTESISD	SSSSSSSSSSSSSSSSSSSSSSSSS------------------	ESLPANGPDSPTVKPPR	3	1		1	1	1		1	
85	TGTVRGDTEPISD	SSSSSSSSSSSSSSSSSSSSSSSSS------------------	ESLPANGPDSPTVKPPR	6	6	2	4					
86	TGTVRGDTESISD	SSSSSSSNSSSSSSSSSSSSSSSSSSS----------------	ESLPANGPDSPTVKPPR	2	1	1		1	1			
36(3D7)	TGTVRGDTEPISD	SSSSSSSSSSSSSSSSSSSSSSSSSSS----------------	ESLPANGPDSPTVKPPR	5	4	1	3					1
37	TGTVRGDTESISD	SSSSSSSSSSSSSSSSSSSSSSSSSSS----------------	ESLPANGPDSPTVKPPR	5	2		2	1	1		2	
87	TGTVRGDTESISD	SSSSSSSSSSSSSSSSSSSSSSSSSSS----------------	ESLPANGLDSPTVKPPR	1	1	1						
88	TGTVRGDTESISD	SISSSSSSSSSSSSSSSSSSSSSSSSS----------------	ESLPANGPDSPTVKPPR	1	1	1						
38	TGTVRGDTEPISD	SSSSSSSSSSSSSSSSSSSSSSSSSSSSS--------------	ESLPANGPDSPTVKPPR	5	1	1		1		1		3
39	TGTVRGDTESISD	SSSSSSSISSSSSSSSSSSSSSSSSSSSS--------------	ESLPANGPDSPTVKPPR	1							1	
40	TGTVRGDTESISD	SSSSSSSSSSSSSSSSSSSSSSSSSSSSS--------------	ESLPANGPDSPTVKPPR	5	4	2	2				1	
42	TGTVRGDTEPISD	SSSSSSSSSSSSSSSSSSSSSSSSSSSSSSS------------	ESLPANGPDSPTVKPPR	3				1	1			2
89	TGTVRGDTESISD	SSSSSSSSSSSSSSSSSSSSSSSSSSSGSSS------------	ESLPANGPDSPTVKPPR	1				1		1		
43	TGTVRGDTESISD	SSSSSSSSSSSSSSSSSSSSSSSSSSSSSSS------------	ESLPANGPDSPTVKPPR	1							1	
46	TGTVRGDTEPISD	SSSSSSSSSSSSSSSSSSSSSSSSSSSSSSSSS----------	ESLPANGPDSPTVKPPR	4	2	1	1				1	1
90	TGTVRGDTESISD	SSSSSSSSSSSSSSSSSSSSSSSSSSSSSSSSS----------	ESLPANGPDSPTVKPPR	1	1	1						
91(CDC1)	TGTVRGDTEPISD	SSSSSSSSSSSSSSSSSSSSSSSSSSSSSSSSSSS--------	ESLPANGPDSPTVKPPR	1	1	1						
49	TGTVRGDTEPISD	SSSSSSSSSSSSSSSSSSSSSSSSSSSSSSSSSSSSS------	ESLPANGPDSPTVKPPR	1								1
50	TGTVRGDTESISD	SSSSSSSISSSSSSSSSSSSSSSSSSSSSSSSSSSSS------	ESLPANGPDSPTVKPPR	1								1
92	TGTVRGDTEPISD	SSSSSSSSSSSSSSSSSSSSSSSSSSSSSSSSSSSSSSS----	ESLPANGPDSPTVKPPR	1	1		1					
93	TGTVRGDTEPISD	SSSSSSSSSSSSSSSSSSSSSSSSSSSSSSSSSSSSSSSSS--	ESLPANGPDSPTVKPPR	1	1	1						
54	TGTVRGDTEPISD	SSSSSSSSSSSSSSSSSSSSSSSSSSSSSSSSSSSSSSSSSSS	ESLPANGPDSLTVKPPR	1							1	
56(FCR3)	-------------	SSSSSSSNSSSSSSSSSSSSSSSSSSSSSSSSSSS--------	ESLPANGPDSPTVKPPR									
				314	172	77	95	54	29	25	55	33

Supplementary Table S1. Primers for PCR amplification and sequencing*.

serine repeat antigen 5 gene (sera5		5' ===> 3'
Primers used for PCR amplification		
1st PCR	sera5-F1	GATAATCCGAAATCTTAAAATGTTACAAA
	sera5-R1	GAGGTACATGATAAATTAAAGATATTATACTACCTTAATAA
	(sera5-F2)	TCCTTGTTTTTCATATTGTGTAAGAATGAA
2nd PCR	sera5-F2	TCCTTGTTTTTCATATTGTGTAAGAATGAA
	sera5-R2	CTACCTTAATAAAATGAATAATGGAGAGTTATGCCCTATT
	(sera5-F3)	TTACGCATACACAAACATTTGTCATTA
Primers used for sequencing		
	sera5-F2	TCCTTGTTTTTCATATTGTGTAAGAATGAA
	sera5-FA	GTTATAAAATGTACAGGAGAAAGTCAAACA
	sera5-FB	GTAAAACTACCATCAAATGGTACAA
	sera5-FC	GATAACAAAGTTGATGTAAGAAAGTATTT
	sera5-FD	GAAAAATGTGATACCTTAGCTTCCA
	sera5-FE	CTTTATCATATGATAACTCAGA
	sera5-FF	GTATGGACCAACTCATTGTCA
	sera5-RA	AATTGGATACAAATAACG
	sera5-RB	TCATCACCACATAAGTTCTG
	sera5-RC	ATACAGCTGCATTACGGAAT
	sera5-RD	GTTATTTACCTGGAATGTCTGA
	sera5-RE	ATACTTTCAGTAGTATCTTTTG,
	sera5-RF	CGTTACATGGACCAGTAACTT

apical membrane antigen 1(ama1)		5' ===> 3'
Primers used for PCR amplification		
1st PCR	ama1-F1	GAAACCTTTACACAAACGTTATACGTACACAGGT
	ama1-R1	CGGCGTAAAAGAAGAATTTACATTATGATGCTT
	(ama1-F2)	CACTTTTGTTAGAGTCTCTTATTAAACGTTAAA
	(ama1-R2)	GCATAAAAGAGAAGCTGATTATATCAGACGTTGA
2nd PCR	ama1-F2	CACTTTTGTTAGAGTCTCTTATTAAACGTTAAA
	(ama1-F3)	CAAATTAATGTACTTGTTATAAATTGTACAAAAATGAGA
	ama1-R2	GCATAAAAGAGAAGCTGATTATATCAGACGTTGA
	(ama1-R3)	CAGACGTTGAAATTATTATAGTTCACATTTTAATAGT
Primers used for sequencing		
	ama1-F3	CAAATTAATGTACTTGTTATAAATTGTACAAAAATGAGA
	ama1-FA	AGAATGCAAAATTCGGATTATGGGT
	ama1-R3	CAGACGTTGAAATTATTATAGTTCACATTTTAATAGT
	ama1-RA	GGTATATCTTCACAATTTCCATCGA

circumsporozoite protein (csp)		5' ===> 3'
Primers used for PCR amplification		
1st PCR	csp-F1	CGTGTAAAAATAAGTAGAAACCACGTATATTAT
	csp-R1	GTTGTTGTTTTTGAGAATAACACATTCTATAAA
	(csp-F2)	GTAGAAACCACGTATATTATAAATTACAATTCAT
	(csp-R2)	GTACAACTCAAACTAAGATGTGTTCTTTATCTA
2nd PCR	csp-F2	GTAGAAACCACGTATATTATAAATTACAATTCAT
	csp-R2	GTACAACTCAAACTAAGATGTGTTCTTTATCTA
	(csp-F3)	CAATTCATGATGAGAAAATTAGCTATTTTATCTGT
	(csp-R3)	CTAAGATGTGTTCTTTATCTAATTAAGGAACAAGA
Primers used for sequencing		
	csp-F3	CAATTCATGATGAGAAAATTAGCTATTTTATCTGT
	csp-FA	AACAATCAAGGTAATGGACAAGGT
	csp-R3	CTAAGATGTGTTCTTTATCTAATTAAGGAACAAGA
	csp-RA	CATCATCATTTTCTCCAAGTGAT

Ca2+-transporting ATPase (serca)		5' ===> 3'
Primers used for PCR amplification		
1st PCR	serca-F0	GCACCTTTTAACAATAGAGAGAATTTTCTTCT
	serca-R1	GCTTTCATAGGAAATATAAGCATCATAGGAAAAT
	(serca-F1)	GGAATTAGTACCCATATATATAATATTATACACACAT
	(serca-F2)	CGTTGAACTTATTATATCTTTGTCATTCGTGAA

	(serca-F3)	CACACATATATGCATAATATTAATATTTATTTATGCTCA
	(serca-R2)	ATAACGTATCGTTTTATTCGGTTGCACCAAA
	(serca-R3)	CCACAAAATAAGAATTTTTGAAAATAGATAAATTAGAACT
2nd PCR	serca-F2	CGTTGAACTTATTATATCTTTGTCATTCGTGAA
	serca-R3	CCACAAAATAAGAATTTTTGAAAATAGATAAATTAGAACT
	(serca-F3)	CACACATATATGCATAATATTAATATTTATTTATGCTCA
	(serca-F4)	GAATGGAAGAGGTTATTAAGAATGCTCATACATA
	(serca-R2)	ATAACGTATCGTTTTATTCGGTTGCACCAAA
	(serca-R4)	CATAACAGCTTAATCAATTTTAATTTTCTTGGTTCTT
Primers used for sequencing		
	serca-F4	GAATGGAAGAGGTTATTAAGAATGCTCATACATA
	serca-FA	TGCTATAGTAAGAAAATTACAAAGTGTT
	serca-FB	TGTATTGTAAAGGTGCACCTGAGA
	serca-FC	TTTAGGGTTCAATCCACCAGAA
	serca-FD	TTTAGGCAAGCACCTTATCTTTAT
	serca-R4	CATAACAGCTTAATCAATTTTAATTTTCTTGGTTCTT
	serca-RA	CCTTAATTTTTCCTGCTGAAAAATATGA
	serca-RB	ATTGTTATTACCTAGTGCTGTTGCT
	serca-RC	CACCTGTAATCATAAATACACGTAT

adenylosuccinate lyase (adsl)		5' ===> 3'
Primers used for PCR amplification		
1st PCR	adsl-F1	ACAACAAATGCATCTCTACCTTTGATAATA
	adsl-R1	GGCGTACATGTTATAAGGTCCTAAATTATA
	(adsl-F2)	GGAAAAACGTAATATAACTCCCCAAAACAA
	(adsl-R2)	GATACCTTAAGACATGGAAATATATACATATATT
2nd PCR	adsl-F2	GGAAAAACGTAATATAACTCCCCAAAACAA
	adsl-R2	GATACCTTAAGACATGGAAATATATACATATATT
	(adsl-F3)	ATTATGGATGTACATGTGAACCAACTGAAAA
	(adsl-R3)	AGTGCCCAACTTGCAGTGTCTTTTTTTATAT
Primers used for sequencing		
	adsl-F3	ATTATGGATGTACATGTGAACCAACTGAAAA
	adsl-FA	CAGATACATTGGCTCGTTTAAA
	adsl-RA	TTATATATTCCTGTGAGAAGTGCT
	adsl-RB	TTGGACATTTCTTTACCAAAGGTA

* In case of amplification failure, primers in brackets were used.

Supplementary Table S2. Accession number of gene sequences used in this study.

gene	number of sequence	Accession numbers
sera5		
Uganda isolates	172	LC580441-LC580612
Burkina Faso isolates	54	LC580613-LC580666
Tanzania isolate	55	AB634928-AB634982
Ghana isolates	33	AB634983-AB635015
ama1		
Uganda isolates	142	LC157527- LC157585, LC580667-LC580749
Tanzania isolate	62	AB715636-AB715697
Ghana isolates	37	AB715698-AB715734
csp1		
Uganda isolates	144	LC580750-LC580893
Tanzania isolate	60	AB502796-AB502855
Ghana isolates	34	AB502856-AB121021
serca		
Uganda isolates	199	LC580894-LC581092
Tanzania isolate	68	AB501575-AB501587, AB501589-AB501643
Ghana isolates	35	AB121058, AB501644-AB501646, AB501648-AB501658, AB501660-AB501678, AB501680
adsl		
Uganda isolates	219	LC157434- LC157526, LC581093-LC581218
Tanzania isolate	68	AB502001-AB502023, AB502025-AB502027, AB502029-AB502042, AB502044-AB502069, AB502071-AB502072
Ghana isolates	35	AB502073-AB502075, AB502078-AB502109

Supplementary Table S3 Polymorphisms in major *P. falciparum* vaccine candidate genes and housekeeping genes in three or four parasite populations from Africa.

	n	No. of polymorphic sites	No. of SNPs	Nucleotide diversity		No. of synonymous sites	No. of non-synonymous sites	Substitutions rate (per site)		dN>dS
				$\theta\pi$ ± SD	θ_S ± SD			dS	dN	P value
A) *sera5* (2562 bp)										
Uganda	172	30	30	0.00023 ± 0.00003	0.00205 ± 0.00037	11	19	0.00025 ± 0.00007	0.00022 ± 0.00009	1.0
Burkina Faso	54	8	8	0.00022 ± 0.00005	0.00069 ± 0.00024	1	7	0.00007 ± 0.00007	0.00026 ± 0.00013	0.1184
Tanzania	55	11	11	0.00021 ± 0.00007	0.00094 ± 0.00028	1	10	0.00007 ± 0.00007	0.00025 ± 0.00008	0.0549
Ghana	33	10	11	0.00032 ± 0.00011	0.00096 ± 0.00030	1	10	0.00000 ± 0.00000	0.00040 ± 0.00015	0.0034
Overall	314	41	42	0.00023 ± 0.00003	0.00253 ± 0.00040	13	29	0.00016 ± 0.00004	0.00025 ± 0.00007	0.1778
B) *ama1* (1866 bp)										
Uganda	142	94	106	0.01407 ± 0.00014	0.00911 ± 0.00094	5	85	0.00199 ± 0.00101	0.01742 ± 0.00223	$<10^{-5}$
Tanzania	62	81	89	0.01364 ± 0.00021	0.00924 ± 0.00103	3	76	0.00194 ± 0.00111	0.01685 ± 0.00162	$<10^{-5}$
Ghana	37	75	85	0.01407 ± 0.00034	0.00963 ± 0.00111	3	70	0.00243 ± 0.00142	0.01729 ± 0.00242	$<10^{-5}$
Overall	241	102	116	0.01405 ± 0.00010	0.00902 ± 0.00089	7	89	0.00206 ± 0.00123	0.01738 ± 0.00243	$<10^{-5}$
C) *csp* (681 bp)										
Uganda	144	40	45	0.00996 ± 0.00035	0.01060 ± 0.00168	2	36	0.00035 ± 0.00022	0.01244 ± 0.00285	0.0007
Tanzania	60	28	32	0.01050 ± 0.00054	0.00882 ± 0.00167	0	25	0.00006 ± 0.00006	0.01320 ± 0.00307	0.0003
Ghana	34	25	29	0.00910 ± 0.00089	0.00898 ± 0.00180	0	29	0.00000 ± 0.00000	0.01144 ± 0.00295	$<10^{-5}$
Overall	238	45	51	0.00998 ± 0.00028	0.01093 ± 0.00163	2	36	0.00023 ± 0.00012	0.01249 ± 0.00326	0.0001
D) *serca+adsl* (5043 bp)										
Uganda	189	64	64	0.00056 ± 0.00003	0.00218 ± 0.00027	23	41	0.00138 ± 0.00071	0.00035 ± 0.00013	1.0
Tanzania	68	37	38	0.00063 ± 0.00004	0.00153 ± 0.00025	17	21	0.00166 ± 0.00067	0.00036 ± 0.00014	1.0
Ghana	35	20	20	0.00048 ± 0.00006	0.00096 ± 0.00022	12	8	0.00151 ± 0.00074	0.00020 ± 0.00009	1.0
Overall	292	98	99	0.00057 ± 0.00002	0.00311 ± 0.00031	41	58	0.00146 ± 0.00064	0.00033 ± 0.00015	1.0

Supplementary Table S4 Haplotype diversity (Hd) of major *P. falciparum* vaccine candidate genes and housekeeping genes in parasite populations from Africa.

A. Hd were analyzed using full-length gene sequences.

	ama1			*csp*			*sera5*			*adsl+serca*		
	n	No. of haplotypes	HD±SD	n	No. of haplotypes	HD±SD	n	No. of haplotypes	HD±SD	n	No. of haplotypes	HD±SD
Tanzania	62	47(47)	0.989 ± 0.005	60	56(54)	0.997 ± 0.004	55	44(42)	0.991 ± 0.006	68	49(21)	0.982 ± 0.008
Ghana	37	32(32)	0.992 ± 0.008	34	29(29)	0.989 ± 0.010	33	31(29)	0.996 ± 0.009	35	19(10)	0.933 ± 0.025
Uganda	142	98(98)	0.991 ± 0.003	144	104(104)	0.993 ± 0.002	172	128(117)	0.994 ± 0.002	189	86(49)	0.964 ± 0.006
Burkina Faso	-	-	-	-	-	-	54	48(48)	0.992 ± 0.007	-	-	-

The number of amino acid sequence variations are shown in parentheses.

B. Hd were analyzed using gene sequences from which insertion/deletion and repeat region were removed.

	ama1			*csp*			*sera5*			*adsl+serca*		
	n	No. of haplotypes	HD±SD	n	No. of haplotypes	HD±SD	n	No. of haplotypes	HD±SD	n	No. of haplotypes	HD±SD
Tanzania	62	47(47)	0.989 ± 0.005	60	39(39)	0.984 ± 0.006	55	10(9)	0.362 ± 0.084	68	49(21)	0.982 ± 0.008
Ghana	37	32(32)	0.992 ± 0.008	34	21(21)	0.943 ± 0.026	33	8(7)	0.472 ± 0.106	35	18(9)	0.926 ± 0.025
Uganda	142	98(98)	0.991 ± 0.003	144	66(65)	0.982 ± 0.003	172	26(16)	0.408 ± 0.048	189	84(47)	0.963 ± 0.006
Burkina Faso	-	-	-	-	-	-	54	9(8)	0.470 ± 0.081	-	-	-

The number of amino acid sequence variations are shown in parentheses.

Supplementary Table S5 Comparison of haplotype diversity (Hd) of SERA5 from Uganda and Burkina Faso trial sites. Those vaccinated with BK-SE36 and control vaccines were compared using the BK-SE36 region, OR and SR regions.

		BK-SE36 region		OR region		SR region	
	n	No. of haplotype	Hd ± SD	No. of haplotype	Hd ± SD	No. of haplotype	Hd ± SD
**Uganda + Burkina Faso**	226	159(144)	0.994+0.002	67(65)	0.898+0.017	77(55)	0.954+0.006
vaccinated	106	85(81)	0.995+0.002	41(39)	0.916+0.021	45(40)	0.958+0.008
controls	120	91(85)	0.991+0.003	44(44)	0.884+0.026	51(40)	0.950+0.009
**Uganda**	172	121(110)	0.992+0.002	49(48)	0.884+0.020	68(51)	0.958+0.007
vaccinated	77	61(58)	0.993+0.004	30(29)	0.890+0.028	40(36)	0.961+0.011
controls	95	73(68)	0.990+0.005	35(35)	0.882+0.029	43(35)	0.955+0.010
**Burkina Faso**	54	46(45)	0.990+0.007	32(32)	0.931+0.026	21(19)	0.905+0.026
vaccinated	29	26(26)	0.990+0.013	20(20)	0.948+0.027	14(13)	0.911+0.033
controls	25	23(22)	0.990+0.016	16(16)	0.897+0.053	14(13)	0.903+0.044

The number of amino acid sequence variations are shown in parentheses.

Supplementary Table S6 Number of serine repeats/amino acid residues in the stretch of serine tandem repeats of the SERA5 SR region from clinical trial sites in Uganda and Burkina Faso.

No. of amino acid	Uganda			Burkina Faso		
	total n=172	vaccinated n=77	controls n=95	total n=54	vaccinated n=29	controls n=25
5	1(0.6)	1(1.3)	N.D.	N.D.	N.D.	N.D.
11	1(0.6)	N.D.	1(1.1)	N.D.	N.D.	N.D.
15	7(4.1)	6(7.8)	1(1.1)	3(5.6)	1(3.4)	2(8.0)
17	3(1.7)	N.D.	3(3.2)	N.D.	N.D.	N.D.
19	1(0.6)	1(1.3)	N.D.	1(1.9)	N.D.	1(4.0)
21	44(25.6)	15(19.5)	29(30.5)	21(38.9)	11(37.9)	10(40.0)
23	21(12.2)	9(11.7)	12(12.6)	10(18.5)	5(17.2)	5(20.0)
25	16(9.3)	7(9.1)	9(9.5)	7(13.0)	6(20.7)	1(4.0)
27	12(7.0)	5(6.5)	7(7.4)	3(5.6)	2(6.9)	1(4.0)
29	7(4.1)	3(3.9)	4(4.2)	1(1.9)	N.D.	1(4.0)
31	43(25.0)	20(26.0)	23(24.2)	5(9.3)	2(6.9)	3(12.0)
33	8(4.7)	5(6.5)	3(3.2)	3(5.6)	2(6.9)	1(4.0)
35	3(1.7)	2(2.6)	1(1.1)	N.D.	N.D.	N.D.
37	1(0.6)	1(1.3)	N.D.	N.D.	N.D.	N.D.
39	2(1.2)	N.D.	2(2.1)	N.D.	N.D.	N.D.
41	2(1.2)	2(2.6)	N.D.	N.D.	N.D.	N.D.
43	N.D.	N.D.	N.D.	N.D.	N.D.	N.D.

The frequency is shown as a percentage in parentheses
